# First Characterization of the Transcriptome of Lung Fibroblasts of SSc Patients and Healthy Donors of African Ancestry

**DOI:** 10.3390/ijms24043645

**Published:** 2023-02-11

**Authors:** Ludivine Renaud, Kristy M. Waldrep, Willian A. da Silveira, Joseph M. Pilewski, Carol A. Feghali-Bostwick

**Affiliations:** 1 Department of Medicine, Rheumatology, Medical University of South Carolina, Charleston, SC 29425, USA; 2 Department of Biological Sciences, School of Life Sciences and Education, Staffordshire University, Stoke-on-Trent ST4 2DF, UK; 3 Department of Medicine, Pulmonary, University of Pittsburgh, Pittsburgh, PA 15213, USA

**Keywords:** systemic sclerosis, scleroderma, pulmonary fibrosis, racial disparity, functional enrichment, pathway analysis

## Abstract

Systemic sclerosis (SSc) is a connective tissue disorder that results in fibrosis of the skin and visceral organs. SSc-associated pulmonary fibrosis (SSc-PF) is the leading cause of death amongst SSc patients. Racial disparity is noted in SSc as African Americans (AA) have a higher frequency and severity of disease than European Americans (EA). Using RNAseq, we determined differentially expressed genes (DEGs; q < 0.1, log2FC > |0.6|) in primary pulmonary fibroblasts from SSc lungs (SScL) and normal lungs (NL) of AA and EA patients to characterize the unique transcriptomic signatures of AA-NL and AA-SScL fibroblasts using systems-level analysis. We identified 69 DEGs in “AA-NL vs. EA-NL” and 384 DEGs in “AA-SScL vs. EA-SScL” analyses, and a comparison of disease mechanisms revealed that only 7.5% of DEGs were commonly deregulated in AA and EA patients. Surprisingly, we also identified an SSc-like signature in AA-NL fibroblasts. Our data highlight differences in disease mechanisms between AA and EA SScL fibroblasts and suggest that AA-NL fibroblasts are in a “pre-fibrosis” state, poised to respond to potential fibrotic triggers. The DEGs and pathways identified in our study provide a wealth of novel targets to better understand disease mechanisms leading to racial disparity in SSc-PF and develop more effective and personalized therapies.

## 1. Introduction

Systemic sclerosis (SSc, aka scleroderma) is a rare autoimmune disease characterized by dysfunction of the immune system, vascular abnormalities, and excessive deposition of extracellular matrix (ECM) components in the skin and internal organs [1]. Amongst SSc patients, 55% of deaths are directly attributed to SSc, and pulmonary fibrosis associated with SSc (SSc-PF) is the leading cause of death [1,2]. To date, only two drugs that slow the progression of lung disease have been approved by the FDA for SSc-PF [3,4]. Thus, more effective therapies are needed. Transcriptomic analysis of human tissues allows the identification of targets for the development of such therapies.

African Americans (AA) have a higher frequency of SSc and worsened disease severities and prognoses than European Americans (EA) [5]. Furthermore, AA exhibit earlier disease onset and are two times more likely to have the diffuse disease subtype than EA, as well as more digital ulcers and pitting, GI tract involvement, and more severe lung and heart disease [6,7,8]. The factors contributing to the higher susceptibility for SSc in AA are poorly understood [5,6,7,8,9,10,11]. 

Given that fibroblasts are the main effectors of fibrosis, we characterized the unique transcriptomic signatures of AA pulmonary fibroblasts in healthy and diseased states [12]. Few studies have focused on the cellular factors underlying severe SSc-PF in AA patients or examined if differences exist between healthy fibroblasts by ancestry. Our extensive collection of cryopreserved primary pulmonary fibroblasts from donors of different ancestries uniquely positioned us to address this gap. Using RNA sequencing (RNAseq) and systems-level analyses, we first compared normal lung (NL) fibroblasts of AA and EA healthy donors by performing an “AA-NL vs. EA-NL” analysis. Next, we defined disease mechanisms in each ancestry by comparing SSc lung (SScL) fibroblasts to NL fibroblasts: “AA-SScL vs. AA-NL” and “EA-SScL vs. EA-NL.” Then, we compared these two lists of differentially expressed genes (DEGs) to reveal unique DEGs in AA-SScL fibroblasts. By comparing DEGs from our first analysis (“AA-NL vs. EA-NL”) to DEGs of our second analysis (“EA-SScL vs. EA-NL”), we examined the possibility of “pre-fibrosis” in AA-NL fibroblasts. Lastly, we identified DEGs in diseased fibroblasts by ancestry with the “AA-SScL vs. EA-SScL” analysis. Selected targets were validated by real-time PCR and immunoblotting. To our knowledge, this is the first characterization of the transcriptome of pulmonary fibroblasts of AA SSc patients and healthy donors. Our goal is to provide insights into the molecular basis for severe PF in SSc patients of African ancestry to identify potential targets and pathways for the development of customized therapies. 

## 2. Results

### 2.1. Perturbation of ECM Organization, Regulation of Myoblast Proliferation, and Histone Methyltransferase Activity in AA-NL Fibroblasts

**Figure 1:**
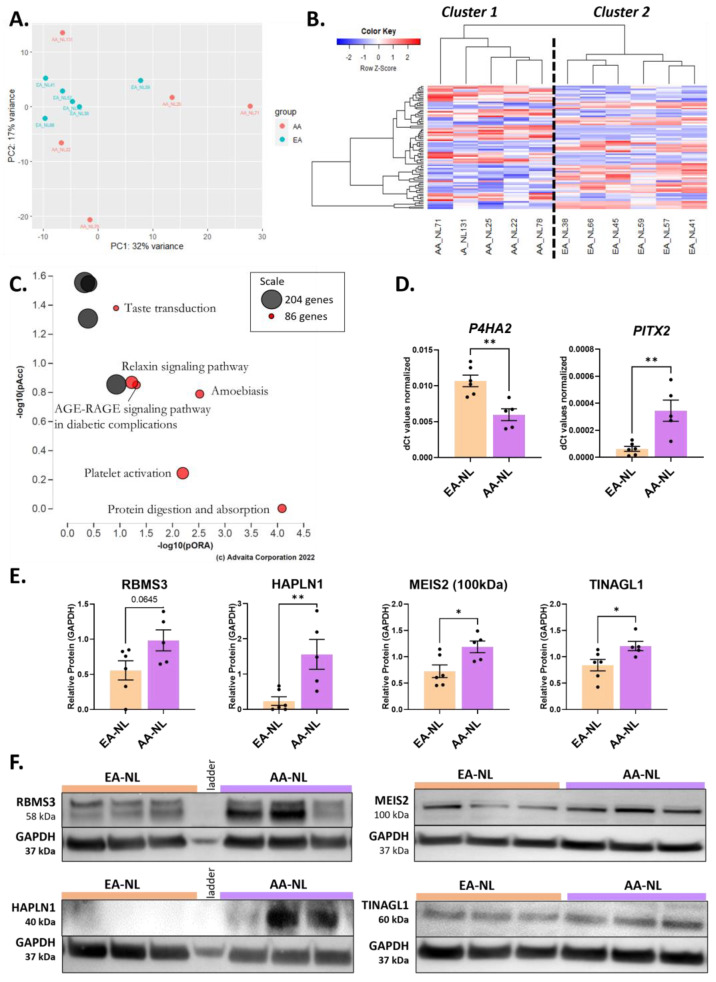
Differential expression analysis between AA-NL and EA-NL. (**A**) PCA plot for “AA-NL vs. EA-NL” DE analysis. (**B**) Heatmap of the genes with a q-value < 0.1 in the “AA-NL vs. EA-NL” DE analysis. (**C**) Perturbation vs. over-representation pathway plot. Dots representing the top 6 impacted pathways are positioned by their *p*-values from two different analyses: the impact analysis (pAcc) vs. the over-representation analysis (pORA). The size of each plot denotes the total number of genes in the corresponding KEGG pathway (for visualization, this number is log(2) scaled). (**D**) Expression levels of *P4HA2* and *PITX2* in AA-NL fibroblasts compared to EA-NL fibroblasts. (**E**) Protein abundance of RBMS3, HAPLN1, MEIS2 (100 kDa band), and TINAGL1 in AA-NL fibroblasts compared to EA-NL fibroblasts, normalized to GAPDH loading control. (**F**) Representative Western blots for each protein mentioned in panel (**E**). For MEIS2, three bands were visible at 60, 65, and 100 kDa, but only the 100 kDa band showed a significant difference in intensity. Unpaired Student’s *t*-Test, * *p* ≤ 0.05, ** *p* ≤ 0.01. Mean ± SEM.

We first examined the transcriptomic signature of AA healthy donors by comparing AA-NL to EA-NL fibroblasts (Figure S1A, DE analysis #1). This DE analysis returned 69 DEGs (Table S1). On the PCA plot (Figure 1A), EA-NL clustered together on both the PC1 (range: −10 to 7) and PC2 axes (range: −2 to 5), suggesting transcriptomic uniformity in this group of healthy fibroblasts. In contrast, AA-NL ranged from −7 to 28 on the PC1 axis and −21 to 14 on the PC2 axis, revealing poor transcriptomic uniformity amongst these fibroblasts and a difference in gene expression signature between healthy AA and EA donors. All the EA-NL and the AA-NL samples clustered by race in the heatmap, as shown by cluster 1 and cluster 2 (Figure 1B). Based on the perturbation versus over-representation pathway plot, the most significant pathways were protein digestion and absorption, platelet activation, amoebiasis, AGE-RAGE signaling pathway in diabetic complications, relaxin signaling pathway, and taste transduction (Figure 1C).

Amongst the 69 DEGs, 36 were more expressed, and 33 were less expressed in AA-NL fibroblasts as compared to EA-NL. To characterize the impact of this transcriptomic signature, a functional enrichment was performed in ToppFun on all downregulated genes and all upregulated genes entered separately (Table 1). Several of the downregulated genes in AA-NL fibroblasts were hits in the query list of pathways and ontologies related to integrin signaling, focal adhesion, collagen biosynthesis and ECM organization, skin development, syndecan-1-mediated signaling events, platelet signaling, and COX reactions. The list of upregulated genes showed enriched GO terms pertaining to histone methyltransferase activity, ECM binding, N-acetyllactosaminide beta-1,6-N-acetylglucosaminyltransferase activity, the binding of activating transcription factor/mRNA 3′-UTR/L27 domain, positive regulation of myoblast proliferation, sensory organ development, and central nervous system projection neuron axonogenesis. The transcript levels of hyaluronan and proteoglycan link protein 1 (*HAPLN1*), meis homeobox 2 (*MEIS2*), RNA binding motif single stranded interacting protein 3 (*RBMS3*), tubulointerstitial nephritis antigen like 1 (*TINAGL1*), paired like homeodomain 2 (*PITX2*), and prolyl 4-hydroxylase subunit alpha 2 (*P4HA2*) were DEGs in AA-NL fibroblasts compared to EA-NL fibroblasts (Table S1). Of these, we validated the downregulation of *P4HA2* and upregulation of *PITX2* by qPCR (Figure 1D) and upregulation of RBMS3, HAPLN1, MEIS2, and TINAGL1 by immunoblotting (Figure 1E,F).

### 2.2. Comparison of Disease Mechanisms in Fibroblasts of AA and EA Patients with SSc

**Figure 2:**
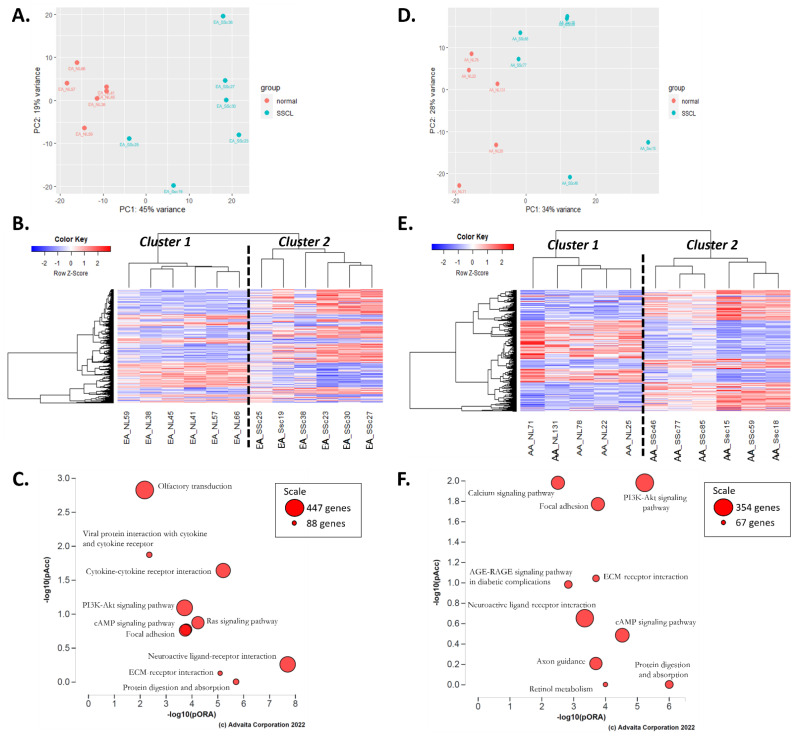
Results of the differential expression analyses “SScL vs. NL” in EA and AA. (**A**) PCA plot for “EA-SScL vs. EA-NL” DE analysis. (**B**) Heatmap of the genes with a q-value < 0.1 in the “EA-SScL vs. EA-NL” DE analysis. (**C**) Perturbation versus over-representation pathway plot for “EA-SScL vs. EA-NL” DE analysis. (**D**) PCA plot for “AA-SScL vs. AA-NL” DE analysis. (**E**) Heatmap of the genes with a q-value < 0.1 in the “AA-SScL vs. AA-NL” DE analysis. (**F**) Perturbation versus over-representation pathway plot for “AA-SScL vs. AA-NL” DE analysis. For panels (**C**,**F**): dots representing the top 10 impacted pathways are positioned by their *p*-values from the impact analysis measuring total perturbation accumulation (pAcc) and from the classic over-representation analysis (pORA). Red: significant combined *p*-values. The size of each dot denotes the total number of genes in the corresponding KEGG pathway (for visualization, this number is log(2) scaled).

In fibroblasts of EA patients with SSc-PF (Figure S1B, DE analysis #2), 2534 DEGs were identified, out of which 1804 DEGs were upregulated, and 730 were downregulated compared to EA-NL fibroblasts. The PCA plot showed good clustering along the PC1 axis with PC1 values below −5 for all EA-NL samples and above −5 for all EA-SScL samples (Figure 2A). On the PC2 axis, EA-NL samples were in a tight cluster spreading from −10 to 10, whereas the EA-SScL samples had a wider range between −20 and 20. EA-SScL and EA-NL samples also clustered well on the heatmap (Figure 2B). Note that EA_SSc-25 had a very distinct signature amongst all other EA-SScL samples and gravitated towards the EA-NL samples on the PCA plot. Based on the perturbation versus over-representation pathway plot, the top 10 most significant pathways were “Protein digestion and absorption,” “ECM-receptor interaction,” “Neuroactive ligand-receptor interaction,” “Focal adhesion,” “cAMP/Ras/PI3K-Akt signaling pathways,” “Cytokine-cytokine receptor interaction,” “Viral protein interaction with cytokine and cytokine receptor,” and “Olfactory transduction” (Figure 2C).

In fibroblasts of AA patients with SSc-PF (Figure S1B, DE analysis #3), only 487 DEGs were identified, out of which 257 were upregulated and 230 downregulated in SSc-PF compared to AA-NL. The PCA plot showed good clustering along the PC1 axis with PC1 values below −5 for all AA-NL samples and above −5 for all AA-SScL samples (Figure 2D). On the PC2 axis, AA-NL samples had a wider range than EA-NL, spreading from −25 to 8, a range similar to AA-SScL samples of −22 and 18. AA-SScL and AA-NL samples clustered well on the heatmap (Figure 2E). Notice, however, the subcluster in the AA-SScL group consisted of samples from three patients with pulmonary hypertension secondary to pulmonary fibrosis (SSc-85, SSc-46, SSc-77 in Figure S2). The top 10 most enriched pathways were “Retinol metabolism,” “Protein digestion and absorption,” “Axon guidance,” “cAMP signaling pathway,” “Neuroactive ligand-receptor interaction,” “AGE-RAGE signaling pathway in diabetic complications,” “ECM-receptor interaction,” “Focal adhesion,” “PI3K-Akt,” and “Calcium signaling” pathways (Figure 2F). 

**Figure 3:**
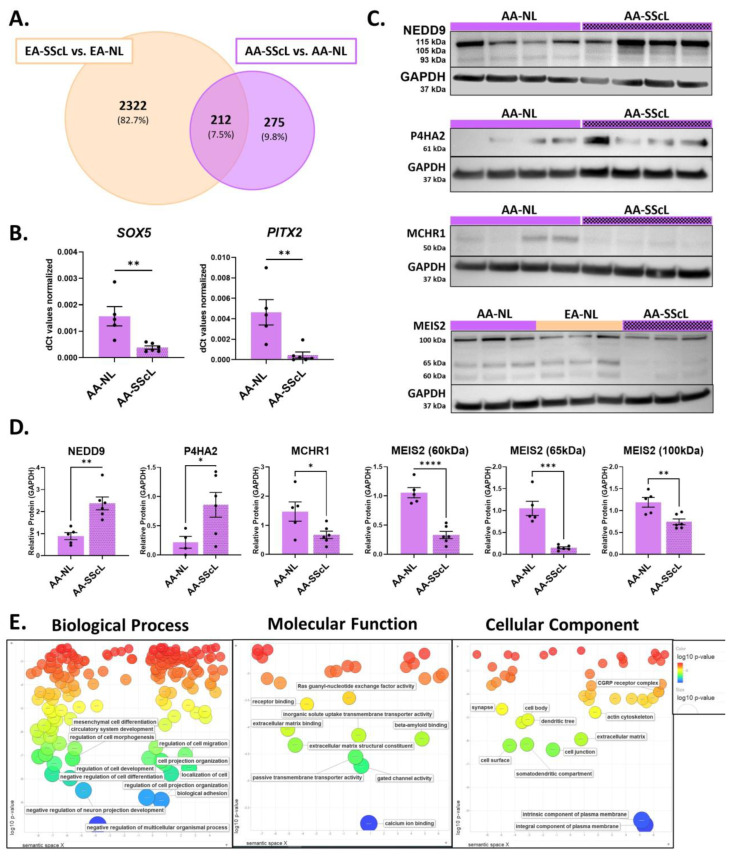
Defining unique disease mechanisms in AA-SScL fibroblasts. (**A**) Venn diagram for the DEGs from the comparisons “SScL vs. NL” in the fibroblasts of SSc-PF AA and EA patients. At the intersect, 212 DEGs are commonly deregulated in both races, while 2322 DEGs are unique to EA-SScL fibroblasts, and 275 DEGs are unique to AA-SScL fibroblasts. (**B**) Expression levels of *SOX5* and *PITX2* in AA-NL and AA-SScL fibroblasts. (**C**) Representative Western blots for NEDD9 (three bands), P4HA2, MCHR1, and MEIS2 (three bands). (**D**) Quantification of protein abundance of NEDD9 (three bands quantified together), P4HA2, MCHR1, and MEIS2 (three bands quantified separately) in the lysates of AA-SScL fibroblasts compared to AA-NL fibroblasts normalized to GAPDH loading control. (**E**) GO terms obtained from ToppFun for the 275 DEGs unique to AA-SScL fibroblasts were entered in REViGO. The scatter bubble plots for Biological Process, Molecular Function, and Cellular Component terms are shown here. Terms in blue and at the bottom of the scatterplot are more enriched than those in red. Only the most enriched terms are labeled in the scatterplot. The bubble color indicates the log10 *p*-value obtained from the ToppFun output. Bubble size indicates the frequency of the GO term in the underlying database, and thus more general terms are larger. The coordinate axes have no intrinsic meaning as REViGO uses multi-dimensional scaling to reduce the dimensionality of a matrix of the GO terms’ pairwise semantic similarities. * *p* ≤ 0.05, ** *p* ≤ 0.01, *** *p* ≤ 0.001, **** *p* ≤ 0.0001. Mean ± SEM.

To determine common disease mechanisms and those exclusive to EA and AA patients, a Venn diagram was created to compare the DE analysis of EA-SSc vs. EA-NL to the DE analysis of AA-SSc vs. AA-NL (Figure S1C). Out of 2809 DEGs entered in the Venn analysis, 2322 (82.7%) were exclusively deregulated in EA-SScL fibroblasts, 212 (7.5%) were commonly deregulated in both EA and AA SScL fibroblasts, and 275 (9.8%) were exclusive to AA-SScL fibroblasts (Figure 3A). We validated several DEGs uniquely deregulated in EA-SScL fibroblasts by qPCR, including the upregulated genes *ACTA2* and *COL1A1,* and the downregulated genes *FASN* and *WNT16* (Figure S3A).

More importantly, the Venn analysis revealed that 275 DEGs were exclusively deregulated in AA-SScL fibroblasts (Table S2), including the downregulated genes *SOX5*, *PITX2* and *MCHR1*, and the upregulated genes *NEDD9* and *P4HA2* that were further validated by qPCR (Figure 3B and Figure S3B). The protein abundance of NEDD9 and P4HA2 was increased in the lysates of AA-SScL fibroblasts, while MCHR1 and MEIS2 were decreased (Figure 3D).

Functional enrichment analysis was conducted using ToppFun on the 275 DEGs that were exclusively deregulated in AA-SScL fibroblasts (Table 2). From this output, the list of GO terms for Biological Process, Molecular Function, and Cellular Component was entered in REViGO (Figure 3E). The most significantly deregulated GO terms (in blue) included “Negative regulation of multicellular organismal process,” “Neuron projection development,” “Biological adhesion,” “Regulation of cell projection organization,” “Calcium ion binding,” and “Integral and intrinsic component of plasma membrane.” In addition, “Cell migration,” “Organization of cell projection,” “Development of circulatory system,” “Differentiation of mesenchymal cells,” “Passive transmembrane transporter activity,” “Gated channel activity,” “Extracellular matrix structural constituent,” “Cell surface and junction,” and “Somatodentritic compartment” were enriched (in green). The pathways and ontologies with the lowest *p*-values and most hit genes in the query list included two ECM-related pathways (“ECM organization” and “Ensemble of genes encoding ECM and ECM-associated proteins”), the molecular function “Calcium ion binding,” and two ontologies pertaining to channel and transporter activity (“Gated channel activity” and “Passive transmembrane transporter activity”) (Table 2).

Using the iPathwayGuide’s impact analysis, we also identified pathways uniquely enriched in AA-SScL fibroblasts, including insulin signaling and diabetes (“Insulin secretion”; “Maturity onset diabetes of the young”; “Type II diabetes mellitus”), TGFβ signaling, and viral infection (“Viral myocarditis”) (Table 2, underlined). The upregulation of BMP5*, GREM1, DCN, and *FBN1*, and the downregulation of PITX2*, TGFΒ3*, and ACVR2B* perturbed the “TGFβ signaling pathway” (Figure S4, * denotes DEGs exclusive to AA-SScL fibroblasts), predicting downstream effects on processes such as iron metabolism, neurogenesis, cell cycle G1 arrest, angiogenesis, ECM neogenesis, immunosuppression, apoptosis induction. The combined enrichment of the “Insulin secretion,” “Maturity onset diabetes of the young,” and “Type II diabetes mellitus” pathway (Figures S5–S7, * denotes DEGs exclusive to AA-SScL fibroblasts) emphasized a strong perturbation of insulin-related processes in AA-SScL fibroblasts resulting from the DEGs MAPK10, IRS2, HHEX, ADCY1*, SLC2A1, CREB3L1*, CACNA1A*, SOCS2, HES1*, RAPGEF4*, CAMK2B, and KCNMB4*.

Additionally, the enrichment of the “Viral myocarditis” pathway (Figure S8), stemming from the strong upregulation of CD55* (aka DAF, encoding a glycoprotein that regulates the complement cascade), would positively regulate the tyrosine kinases ABL1/2 and the small GTP-binding proteins RAC1/2/3, leading to actin reorganization. Finally, the enrichment of the “Proteoglycans in cancer” pathway (Figure S9) was so profound that impact analysis predicted an overall perturbation of the pathway affecting downstream outcomes such as inhibition of angiogenesis, proliferation and survival, cell growth and survival, and apoptosis. This perturbation resulted from the upregulation of IGF2, KDR*, TLR4*, TWIST2, COL1A1, COL1A2*, DCN, and *LUM,* and the downregulation of HPSE2* and *CAMK2B*. 

**Figure 4:**
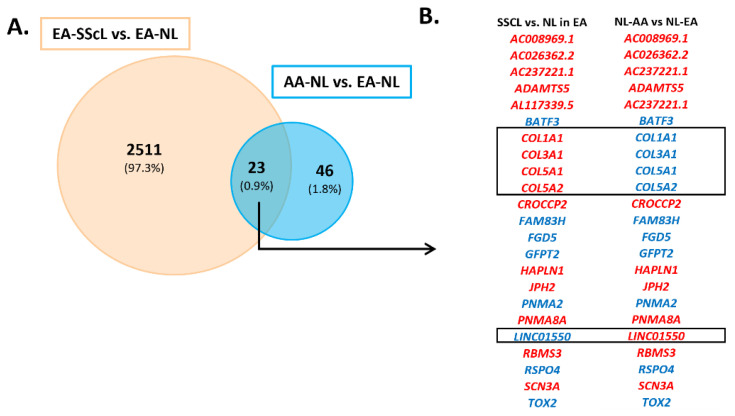
Functional enrichment of the DEGs unique to AA-SScL fibroblasts. (**A**) Venn diagram comparing the DEGs from the comparison “EA-SScL vs. EA-NL” to the DEGs obtained from the comparison “AA-NL vs. EA-NL.” At the intersect, 23 DEGs are commonly deregulated in AA-NL and EA-SScL fibroblasts, out of which 18 are deregulated in the same direction (**B**). Red: upregulated DEGs, blue: downregulated DEGs. The boxes in panel B show the DEGs at the intersect of the Venn diagram in panel A that are oppositely deregulated: *COL1A1, COL3A1, COL5A1, COL5A2, LINC01550*.

We were intrigued by the detection of more DEGs in EA-SScL vs. EA-NL fibroblasts (2511 DEGs) than in AA-SScL vs. AA-NL fibroblasts (487 DEGs), as well as the 69 DEGs in AA-NL compared to EA-NL fibroblasts. Therefore, we examined the possibility of a disease-like signature in AA-NL by comparing the 69 DEGs in AA-NL fibroblasts to the 2511 DEGs in EA-SScL fibroblasts (Figure 4A and Figure S1D). We identified 23 DEGs commonly deregulated (intersect) in AA-NL and EA-SScL fibroblasts (0.9% of all genes entered in this Venn analysis), out of which 18 had the same direction of regulation (Figure 4B): *ADAMTS5, CROCCP2, HAPLN1, JPH2, PNMA8A, RBMS3, SCN3A*, and four not-yet-defined nucleotide sequences were upregulated, and *BATF3, FAM83H, FGD5, GFPT2, PNMA2, RSPO4,* and *TOX2* were downregulated. HAPLN1 and RBMS3 were quantified by immunoblotting in the lysates of AA-NL and EA-NL fibroblasts (Figure 1E,F). Note that *COL1A1, COL3A1, COL5A1, COL5A2,* and *LINC01550* were deregulated in the opposite direction of regulation in AA-NL and EA-SScL fibroblasts (box in Figure 4B). Together our data suggest that AA-NL fibroblasts have a transcriptomic signature that holds several characteristics reminiscent of the pathological signature observed in EA-SScL fibroblasts, suggesting a propensity for molecular activation which may explain some of the racial disparity observed in SSc-PF.

### 2.3. AA-SScL vs. EA-SScL: What Are the Differences in Disease State?

**Figure 5:**
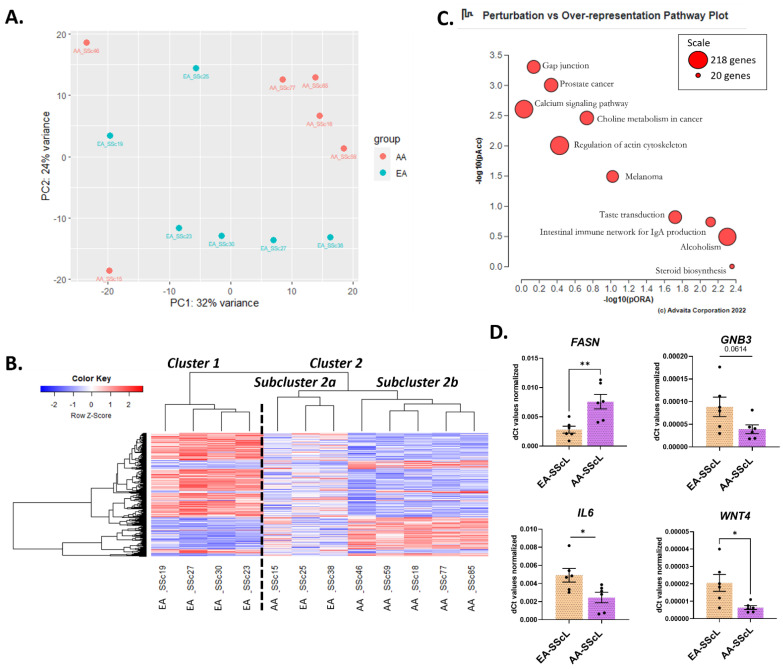
Characterizing the differences in disease state between AA and EA SScL fibroblasts. (**A**) PCA plot for “AA-SScL vs. EA-SScL” DE analysis. (**B**) Heatmap of the genes with a q-value < 0.1 in the “AA-SScL vs. EA-SScL” DE analysis. (**C**) Perturbation vs. over-representation pathway plot for “AA-SScL vs. EA-SScL.” Dots representing the top 10 impacted pathways are positioned by their *p*-values from 2 different analyses: the impact analysis (pAcc) vs. over-representation analysis (pORA). The size of each plot denotes the total number of genes in the corresponding KEGG pathway (for visualization, this number is log(2) scaled). (**D**) Validation of selected genes by qPCR in EA-SScL and AA-SScL samples. * *p* ≤ 0.05, ** *p* ≤ 0.01. Mean ± SEM.

The SScL fibroblasts in this study were cultured from the explanted lungs of SSc patients with advanced PF who underwent transplantation. We compared the transcriptome of SSc-PF in AA and EA patients. The DE analysis AA-SScL vs. EA-SScL returned 384 DEGs, including 109 upregulated and 275 downregulated genes (Figure S1E, DE analysis #4). The PCA plot (Figure 5A) showed poor clustering of the AA and EA SScL samples. However, two main clusters were defined on the heatmap (Figure 5B). Cluster 1 contained four EA samples (SSc19, SSc27, SSc30, SSc23) with fibrosis (FVC < 70%) and no pulmonary hypertension (PH), barring SSc19 with mild PH (pulmonary artery pressure mean = 26 mm Hg) (Figure S2, Table S3). Cluster 2 was divided into two subclusters: 2a and 2b. Subcluster 2a consisted of one AA sample (SSc15) and two EA samples (SSc25, SSc38) from patients who had extensive pulmonary fibrosis (FVC < 70%) without evidence of PH. The transcriptomic signatures of these samples were less defined than cluster 1 and subcluster 2b. Subcluster 2b contained five AA samples (SSc46, SSc59, SSc18, SSc77, SSc85) from patients who had both pulmonary fibrosis and PH (FVC ranging from 18−46%, and PA mean >25 mm Hg), except for SSc18 who just had pulmonary fibrosis. Their transcriptomic signatures were markedly distinct from cluster 1.

The perturbation versus over-representation pathway analysis showed that the most significant pathways in AA-SScL fibroblasts were “Calcium signaling,” “Regulation of actin Cytoskeleton,” “Gap junction,” “Prostate cancer,” “Choline metabolism in cancer,” “Melanoma,” “Taste transduction,” “Intestinal immune network of IgA production,” “Alcoholism,” and “Steroid biosynthesis” (Figure 5C).

Of the 384 DEGs identified in the DE analysis “AA-SScL vs. EA-SScL,” only 286 were found in ToppFun, meaning that 98 DEGs are novel transcripts/uncategorized genes. ToppFun returned the enrichment shown in Table 3. Pathways and ontologies related to the biosynthesis of steroid hormones (“Steroid biosynthesis” and “Activation of gene expression by SREBF”), “Alcohol metabolic process,” “Genes encoding ECM,” “Beta1 integrin cell surface interactions,” and “Neutrophil activation” were enriched in AA-SScL fibroblasts compared to EA-SScL. The impacted pathways are underlined in Table 3. Downregulated *PDGFB* contributed to the enrichment of the “Gap junction” and “Calcium signaling” pathways (Figures S10 and S11). This was predicted to perturb MAPK signaling, leading to upregulation of connexins *GJA1* and *GJD2* in the “Gap junction” pathway and downregulation of receptor tyrosine kinase (*RTK*), *ERBB2/3/4, EGFR, PDGFRA/B,* and *PLCG1/2* in the “Calcium signaling” pathway. Combined downregulation of the receptor *GABBR1* and the G protein *GNB3* enriched the “GABAergic synapse” pathway, predicting downstream upregulation of adenylate cyclases 1 through 9 (*ADCY1-9*) and the calcium voltage-gated channel subunits 1A and 1B (*CACNA1A/1B*) (Figure S12). Downregulation of *PDGFB* and *MYH1* and upregulation of *ITGA8* and *FGF5* enriched the “Regulation of actin cytoskeleton” pathway (Figure S13). iPathwayGuide predicted a subsequent decrease in the *PDGFB-RTK-Sos-Ras-PI3K* subpathway with an enhancement of the *ITGA8-FAK-Cas-CrKII* axis, upregulating the dedicator of cytokinesis 1 (Dock180) gene and genes encoding small GTPase RAC proteins. Finally, downregulated *PABPC1L/4L*, encoding polyadenylate-binding protein cytoplasmic-like-1 and -4, enriched the “RNA degradation” pathway, specifically “mRNA surveillance” and “Cytoplasmic deadenylation Ccr4-NOT complex” (Figure S14). This may downregulate *CNOT7/8* and *PAN3*.

We validated 4 DEGs by qPCR, confirming the upregulation of fatty acid synthase (*FASN*) and the downregulation of G-protein subunit beta 3 (*GNB3*), interleukin 6 (*IL6*), and Wnt family member 4 (*WNT4*) (Figure 5D). 

## 3. Discussion

SSc patients with African ancestry have an earlier age of disease onset, a higher probability of being diagnosed with diffuse SSc and severe pulmonary fibrosis, and greater morbidity and mortality than EA patients [11]. Despite this known racial disparity in SSc, few studies have focused on AA patients, possibly due to lack of access to AA lung tissues. For the first time, we characterized the unique transcriptomic signature of AA fibroblasts, not only in disease but also in a healthy state, providing a wealth of novel DEGs and pathways to consider when developing personalized therapies for SSc-PF. Our results are summarized as follows: (A) In AA-NL fibroblasts compared to EA-NL fibroblasts, 69 DEGs were identified, emphasizing perturbation of ECM organization, regulation of myoblast proliferation, and histone methyltransferase activity in AA-NL fibroblasts. (B) Considering disease mechanisms by ancestry, the DE analysis of “EA-SScL vs. EA-NL” yielded 2534 DEGs, whereas the “AA-SScL vs. AA-NL” analysis only returned 487 DEGs. Amongst the top 10 most enriched pathways in AA-SScL fibroblasts were “Retinol metabolism,” “Protein digestion and absorption,” “Axon guidance,” “cAMP signaling pathway,” “Neuroactive ligand-receptor interaction,” “AGE-RAGE signaling pathway in diabetic complications,” “ECM-receptor interaction,” “Focal adhesion,” “PI3K-Akt,” and “Calcium signaling” pathways. (C) Comparing the disease mechanisms “SScL to NL” in both AA and EA fibroblasts, only 212 DEGs (7.5%) overlapped, highlighting that disease mechanisms are distinct by ancestry. (D) Two hundred and seventy-five DEGs were exclusive to AA-SScL fibroblasts, causing the enrichment of pathways pertaining to insulin signaling and diabetes, TGFβ signaling, ECM organization, adhesion, the development of neuron projection, calcium ion binding along with channel and transporter activity to name a few. *SOX5, PITX2, MCHR1, NEDD9,* and *P4HA2* were genes of interest that we validated by qPCR and immunoblotting. These unique disease mechanisms may explain racial disparities in AA patients. (E) Comparison of the DEGs from “AA-NL vs. EA-NL” to the ones from “EA-SScL vs. EA-NL” revealed 18 DEGs with the same differential expression, suggesting that AA-NL fibroblasts have a disease-like signature reminiscent of EA-SScL fibroblasts and thus may be in a “pre-fibrotic” state, poised to respond to fibrotic triggers. (F) Comparison of “AA-SScL vs. EA-SScL” revealed 384 DEGs and the enrichment of “Calcium signaling,” “Regulation of actin Cytoskeleton,” “Gap junction,” “Prostate cancer,” “Choline metabolism in cancer,” “Melanoma,” “Taste transduction,” “Intestinal immune network of IgA production,” “Alcoholism,” and “Steroid biosynthesis” pathways. 

Together, our results reveal transcriptomic differences between pulmonary fibroblasts of EA and AA individuals–healthy and diseased–and suggest that disease mechanisms differ considerably by ancestry. This may contribute to a better understanding of the racial disparity in SSc-PF and the development of more effective therapeutic strategies for AA patients. 

### 3.1. The Unique Transcriptome of AA-NL Fibroblasts (AA-NL vs. EA-NL)

Some of the 69 DEGs identified in the “AA-NL vs. EA-NL” analysis have well-defined roles in fibrogenesis, while others remain uncharacterized. Two genes involved in TGFβ and Wnt signaling pathways, both associated with the development of PF in SSc patients [13,14], were differentially expressed in AA-NL fibroblasts. The disintegrin and metalloproteinase with thrombospondin motifs 5 (*ADAMTS5*) was upregulated in AA-NL, and this gene encodes a critical enzyme for versican and aggrecan processing [15], able to modulate tissue repair by degrading pericellular aggrecan and promoting TGFβ signaling in fibroblastic cells [16]. Another gene, the TraB domain containing 2A (*TRABD2A*) was downregulated in AA-NL. This metalloproteinase acts as a negative regulator of the Wnt signaling pathway, which is abnormally activated in fibrosis [14]. Specifically, elevated nuclear β-Catenin, a downstream factor of Wnt signaling, is increased in SSc fibrotic lung tissues [17]. TRABD2A cleaves the eight N-terminal residues of a subset of Wnt proteins, including WNT3A and WNT5 [18]. Interestingly, WNT3A has been shown to activate β-Catenin and induce profibrotic gene expression in human fibroblasts [19]. Reduced *TRABD2A* expression in AA-NL may imply a decreased ability to inactivate Wnt signaling, a pathway associated with fibrosis. Thus, the upregulation of *ADAMTS5* and downregulation of *TRABD2A* contributed to the enhancement of the TGFβ and Wnt signaling pathways in AA-NL fibroblasts.

Processes and pathways pertaining to collagen biosynthesis and ECM organization are markedly perturbed in AA-NL fibroblasts by (1) the downregulation of *LAMC1, P4HA2, FILIP1,* and *EMP1,* and (2) the upregulation of *TINAGL1* and *PITX2. LAMC1* encodes the ECM glycoprotein laminin subunit gamma-1, a component of basement membranes that acts as a cellular scaffold in tissues, and its downregulation in AA-NL fibroblasts is consistent with the decrease detected in IPF lungs by quantitative proteomic characterization [20]. *FILIP1* encodes filamin-A interacting protein 1, an actin cross-linking protein that degrades filamin-A (FLNA) [21] and plays a role in the phagocytosis and intracellular digestion of collagen from the cell surface [22]. *EMP1* encodes epithelial membrane protein 1, which is involved in cell proliferation and migration, ECM organization, and cell-cell and cell-ECM interactions [23,24]. *EMP1* is upregulated in proliferating fibroblasts [25], and TGFβ2 induces miR-31 in dermal wound healing, which downregulates *EMP1* and promotes keratinocyte proliferation and migration [26]. *TINAGL1* encodes tubulointerstitial nephritis antigen-like 1, an ECM glycoprotein that interacts with both structural matrix proteins and cell surface receptors [27]. In *Sma-GFP* transgenic mice, Tinagl1 was amongst the most upregulated glycoproteins expressed in skin myofibroblasts and was linked to myofibroblast-mediated wound healing [28]. Additionally, TINAGL1 can activate the TGFβ signaling pathway and increase VEGF secretion, a mediator of SSc vasculopathy [29,30,31]. *PITX2* encodes paired-like homeodomain transcription factor 2. It induces procollagen-lysine, 2-oxoglutarate 5-dioxygenase (PLOD) expression, which catalyzes the hydroxylation of lysyl residues in pre-pro-collagen, leading to collagen cross-linking and deposition [32]. Interestingly, SSc dermal fibroblasts have increased *PLOD* expression levels. However, despite upregulation of *PITX2* in our AA-NL fibroblasts, we did not observe *PLOD* upregulation [33]. Both the TGFβ and Wnt signaling pathways regulate *PITX2* [34,35,36], two pathways that are central in the development of PF in SSc patients [13,14]. The downregulation of *P4HA2, FILIP1* and *EMP1* and the upregulation of *TINAGL1* and *PITX2* may contribute to altered collagen biosynthesis and ECM organization in AA-NL fibroblasts. 

Both *PITX2* and *MEIS2* were upregulated hit genes in the enrichment of “positive regulation of myoblast proliferation” in AA-NL, a biological process observed during lung fibrosis [37]. *MEIS2* is a transcriptional regulator whose role in fibrosis has not been studied; however, it has been characterized in embryonic development and cancer. For instance, conditional *Meis2* knockout mice exhibit abnormal craniofacial skeleton, cartilage, heart, and cranial nerve development [38]. Similarly, MEIS2 participates in avian lung morphogenesis whereby it and TGFβ2 are induced by retinoic acid stimulation [39]. In colorectal cancer, MEIS2 promotes cell migration and invasion [40]. In HepG2 cells, MEIS2 cooperatively activates genes in a complex with PBX1 and KLF4, a transcription factor whose expression is decreased in SSc skin fibroblasts and has been implicated in fibrosis [41,42,43]. 

The transcriptome of AA-NL fibroblasts also showed a signature towards enhancement of inflammation and immunity with the downregulation of *ABCG1* and *MICA* and the upregulation of *EPHB1. ABCG1* encodes for ATP-binding cassette sub-family G member 1, an ABC transporter known to play critical roles in pulmonary homeostasis and regulate inflammatory responses [44]. *ABCG1* deficiency has been shown to promote lipid accumulation, proinflammatory macrophage activation, and pulmonary fibrosis [44,45,46]. Deletion of *Abcg1* in mice exacerbates pulmonary fibrosis in response to bleomycin challenge [47]. *MICA* encodes MHC class I polypeptide-related sequence A, a highly polymorphic cell surface glycoprotein that can activate T cells and natural killer (NK) cells through the KLRK1/NKG2D receptor [48]. In patients with advanced hepatic fibrosis, *MICA* expression was significantly lower than in patients with no or mild hepatic fibrosis [49]. TGFβ1 has been shown to suppress *MICA* expression in hepatic stellate cells [49]. *EPHB1* encodes a receptor tyrosine kinase that binds promiscuously transmembrane ephrin-B family ligands residing on adjacent cells, leading to contact-dependent bidirectional signaling into neighboring cells [50]. Recently, the role of ephrin receptors and their ligands in immunity and the development of organ fibrosis has been described [51]. Although the function of EPHB1 remains unclear, EPHB2 has been shown to mediate the trafficking of ephrin-B-expressing macrophages to the liver, initiating the inflammatory response that stimulates the differentiation of hepatic stellate cells into fibrogenic myofibroblasts, leading to the development of fibrosis [52]. Cleavage of EPHB2 by ADAM10 in fibroblasts induces their activation and increases skin and lung fibrosis [53]. Taken together, the downregulation of *ABCG1* and *MICA* and upregulation of *EPHB1* may enhance inflammation and immunity in AA-NL fibroblasts and predispose these fibroblasts to fibrosis [54]. 

Loss of microvessels and altered angiogenesis are hallmarks of SSc [31,55], and two genes, *HTATIP2* and *RASIP1,* encoding proteins that have angiogenic properties, were downregulated in AA-NL fibroblasts. *HTATIP2* encodes an oxidoreductase that may act as a redox sensor linked to transcription by regulating nuclear import [56]. Its isoform 1 has proapoptotic and antiangiogenic properties on tumor cells, while isoform 2 is only antiapoptotic [56]. Noticeably, *HTATIP2* is also downregulated in IPF fibroblasts [57]. *RASIP1* encodes Ras-interacting protein 1, which is required for the proper formation of vascular structures developed by vasculogenesis and angiogenesis [58]. Koo et al. [59] proposed that in the absence of *RASIP1*, RhoA activity is increased, causing remodeling defects that lead to reduced vessel outgrowth and activation of the ROCK pathway that promotes SSc-PF [60]. *RASIP1* is significantly downregulated in patients with SSc-ILD [61].

The DEGs of the “AA-NL vs. EA-NL” analysis are involved in numerous cellular processes, including TGFβ and Wnt signaling, collagen biosynthesis and ECM organization, inflammation and immunity, COX reactions and platelet activation, loss of microvessels and altered angiogenesis, cellular metabolism, and calcium oscillations. These transcriptomic differences highlight that variation exists between healthy AA and EA fibroblasts. 

### 3.2. The Transcriptome of AA-NL Fibroblasts Has Disease-Like Features

Surprisingly, 18 of the 69 DEGs identified in AA-NL fibroblasts were commonly deregulated in EA-SScL fibroblasts. The transcription factor *BATF3* (aka *SNFT*) was downregulated, and it is known to repress MMP1 [62], a collagenase that breaks down collagen type I, II, and III [63]. Another downregulated gene, *RSPO4*, encodes R-spondin 4, a regulator of the Wnt signaling pathway, which is often perturbed in various fibrotic disorders, including SSc [64,65]. The upregulated *HAPLN1* encodes a hyaluronan and proteoglycan link protein involved in collagen organization and immune cell infiltration in dermal fibroblasts [66] and the modulation of myofibroblasts in pulmonary fibrosis [67]. *RBMS3*, an RNA-binding protein of the *C-MYC* gene single-strand binding protein family, was upregulated in AA-NL and EA-SScL fibroblasts. It is also upregulated in SSc lung, and when associated with WNT5A and MSI2, it can influence lung outcomes in SSc-PF [68]. RBMS3 has also been shown to inhibit microvessel formation by downregulating MMP2 and β-Catenin [69]. This is interesting since the loss of microvessels and altered angiogenesis are hallmarks of SSc [31,55]. *FGD5* was downregulated in AA-NL and EA-SScL fibroblasts. FGD5 is a factor known to regulate neovascularization and the coupling of PI3 kinase to activate VEGFR2 in the early endosome compartment and to retain VEGFR2 in recycling endosome compartments [70,71].

Although fibroblasts are considered non-excitable cells, studies have focused on their electrophysiological properties in association with cardiomyocytes and cardiac disease [72,73]. Recently, the signaling mechanisms regulating gene expression in human fibroblasts have been investigated, showing that calcium oscillations play a central role in triggering proliferation, production of ECM and TGFβ, and myofibroblast transformation [74,75]. Two relevant genes, *JPH2* and *SCN3A*, were commonly upregulated in AA-NL and EA-SScL fibroblasts. Junctophilin 2 (*JPH2*) is a membrane-binding protein that provides a structural bridge between the cell surface and calcium release channels [76]. Following cleavage and release from the membrane, JPH2 can translocate to the nucleus, bind DNA, and repress genes implicated in cell growth and differentiation, hypertrophy, inflammation, and fibrosis in cardiomyocytes [77]. The sodium voltage-gated channel alpha subunit 3 gene (*SCN3A*) encodes the I_NA.TTX_ voltage-gated Na^+^ ion channel, which is expressed in human cardiac fibroblasts and may participate in fibroblast-myocyte electrical coupling [78]. Intriguingly, SCN3A was identified as a potential marker in patients with chronic thromboembolic pulmonary hypertension, and it is predicted to participate in the ion flux of pulmonary artery smooth muscle cells (PASMC), which modulates excitation and contraction of pulmonary vessels [79]. Given the significance of pulmonary arterial hypertension in SSc [80], SCN3A warrants further consideration. Moreover, JPH2 and SCN3A both present interesting opportunities for future investigation, as their functions in fibroblasts and fibrosis remain unexplored.

Cell metabolism in SSc is an emerging field of study [81]. *GFPT2* (aka *GFAT2*) was downregulated in AA-NL and EA-SScL fibroblasts and encodes glutamine-fructose-6-phosphate transaminase 2, the rate-limiting enzyme that controls the flux of glucose into the hexosamine biosynthetic pathway [82]. This pathway is essential for protein glycosylation and GlcNAcylation, with roles in nutrient sensing, stress responses, and cell growth [83,84]. It additionally induces ECM overproduction when glucose levels are high [85]. In fact, the hexosamine biosynthetic pathway is characterized as a profibrotic pathway [83], and overexpression of GFPT2 can induce Tgfβ expression in mouse embryonic fibroblasts in vitro [86]. In contrast, inhibition of GFPT2 expression can prevent the expression of biologically active TGFβ1 [81]. 

Taken together, our findings suggest that the gene signature in AA-NL fibroblasts shares some of the functional perturbation captured in EA-SScL fibroblasts. This may suggest that fibroblasts from AA individuals are poised to respond to fibrotic triggers based on their activated molecular profile, suggesting that AA individuals are predisposed to fibrogenic disorders and SSc-PF. 

### 3.3. Disease Mechanisms Unique to AA-SScL

The transcriptomes of SScL fibroblasts in AA and EA patients shared only 7.5% of DEGs, revealing that mechanisms of SSc-PF progression are quite different in AA and EA fibroblasts. The 275 DEGs unique to AA-SScL fibroblasts contributed to the ToppFun functional enrichment of ECM-related pathways and of the Molecular Function GO terms “Calcium ion binding,” “Gated channel activity,” and “Passive transmembrane transporter activity.”

Additionally, the iPathwayGuide impact analysis showed a strong perturbation of the “TGFβ signaling pathway” in AA-SScL fibroblasts driven by the upregulation of *BMP5, GREM1, DCN,* and *FBN1* and the downregulation of *PITX2, TGFΒ3,* and *ACVR2B*. *DCN* encodes decorin, one of the most abundant proteoglycans of the ECM secreted by fibroblasts [87]. SSc dermal fibroblasts overexpress decorin [88], but the literature suggests it is a double-edge sword in fibrogenesis. On one end, decorin is known for its anti-fibrotic properties due to its ability to form a complex with TGFβ, thus preventing activation of TGFβ receptors and the Smad pathway [89,90]. The combined upregulation of *DCN* and downregulation of *TGFΒ3* in AA-SScL reflects the reported anti-fibrotic properties of the proteoglycan DCN, an endogenous inhibitor of TGFβ, that results in *TGFΒR2* downregulation. On the other end, in its soluble form, decorin is an endogenous ligand to TLR2 and TLR4 and can activate the p38 MAPK and ERK pathways, leading to pro-inflammatory signaling; it may also promote lung fibrosis when degraded by cathepsin-S because fragmentation disrupts its anti-fibrotic capabilities [91,92,93]. Thus, decorin may act as a damage-associated molecular pattern (DAMP) [91]. 

The DE analysis of “AA-SScL vs. AA-NL” returned *TGFΒ3* as a downregulated gene, indicating its expression is higher in AA-NL fibroblasts than AA-SScL fibroblasts, a surprising observation. Reports of TGFβ3 function in fibrosis are conflicting since some studies report that it negatively regulates the fibrotic response and accelerates wound healing without scarring [94,95]. However, in vitro experiments show that all three TGFβ isoforms exert similar fibrogenic effects [96]. The “TGFβ signaling pathway” was also enriched by the upregulation of *BMP5*, despite the high expression levels of *GREM1* (a known inhibitor of BMPs), potentially causing the upregulation of the receptors *BMPR2* and *AMHR2*. The overall perturbation of this pathway has far-reaching effects on multiple processes.

The impact analysis also revealed the enrichment of the “Type II diabetes mellitus” pathway resulting from the upregulation of *MAPK10* and *IRS2* and the downregulation of *CACNA1A* and *SOCS2*, a signature that may upregulate the insulin receptor (*INSR*), *PI3K,* and insulin resistance. Likewise, both the “Maturity onset diabetes of the young” and “Insulin secretion” pathways are exclusively enriched in AA-SScL fibroblasts. In the “Maturity onset diabetes of the young” pathway, the upregulation of *HHEX* and downregulation of *HES1* could increase the expression of *NEUROG3* and outcomes such as the Insulin signaling pathway. The “Insulin secretion” perturbation showed upregulated *CREB3L1* (aka *OASIS*), a transcription factor involved in unfolded protein response that could enhance insulin secretion by promoting its release from secretory granules. CREB3L1 can also induce genes involved in ECM formation [97]. Together, these three enriched pathways highlight an overall perturbation of insulin-related processes and insulin resistance in AA-SScL fibroblasts. 

The “Proteoglycans in cancer” pathway was also exclusively enriched in AA-SScL fibroblasts, driven by the upregulation of *IGF2, DCN, LUM, COL1A1, COL1A2,* and the receptors *KDR* and *TLR4*. The perturbation caused by this signature was predicted to affect angiogenesis, cell growth, proliferation, survival, apoptosis, stress fiber formation, cell adhesion and spreading, cell migration, and invasion. We previously reported that *IGF2* is upregulated in SScL fibroblasts and promotes fibrosis by inducing *COL1A1*, *TGFβ2*, and *TGFβ3* through activation of the phosphatidylinositol-3 kinase and Jun N-terminal kinase signaling cascades after binding to the receptors IGF1R, IR and the IGF1R/IR hybrid [98,99]. In SSc pulmonary and dermal tissues, levels of *TLR4* and its damage-associated ligands are elevated, increasing sensitivity to TGFβ1 and enhancing the Smad pathway, fibroblast activation, and persistent fibrogenesis [100]. As discussed under the “TGFβ signaling pathway,” soluble decorin is an endogenous ligand to TLR2 and TLR4 and can activate the p38 MAPK and ERK pathways, promoting inflammation and lung fibrosis and acting as a DAMP [91,92,93]. Further investigation is required to fully characterize the outcome of the combined upregulation of *DCN* and *TLR4* observed exclusively in the disease mechanisms of AA-SScL fibroblasts. Overall, the enrichment of the “Proteoglycans in cancer” pathway in AA-SScL fibroblasts highlight a deep deregulation of several processes tied to persistent fibrosis. 

The gene *NEDD9* was uniquely upregulated in AA-SScL fibroblasts in association with the biological process of cell adhesion. On immunoblots, differential phosphorylation of NEDD9 results in three bands: 93 kDa (phosphorylation at a single serine residue), 105 kDa (serine-threonine phosphorylation), 115 kDa (serine, threonine, and tyrosine phosphorylation) [101,102]. We observed upregulation of all three forms. NEDD9 has been described as a critical marker of the transition from adaptative to pathogenic fibrosis that increases COL3A1 expression levels by stabilizing the physical interaction between NKX2-5 and the COL3A1 promoter in human pulmonary artery endothelial cells [103]. Our data also showed that *COL3A1*, a type III collagen known to regulate type I collagen fibril formation [104], was commonly upregulated in AA-SScL and EA-SScL fibroblasts relative to their respective NL counterparts. Since type III collagen regulates cell adhesion, migration, proliferation, and differentiation of cells [105], processes that are enriched in AA-SScL, targeting the NEDD9-COL3A1 axis for inhibition, may have far-reaching effects on fibrogenesis. Indeed, Nedd9 inhibition has been shown to prevent pulmonary arterial hypertension (PAH) in *Nedd9*-deficient transgenic mice [103]. *P4HA2* is a key enzyme in collagen synthesis that was uniquely upregulated in AA-SScL fibroblasts. We already mentioned this gene in the discussion Section 3.1 as it was downregulated in AA-NL fibroblasts. When present in excess, P4HA2 enhances the deposition of collagens and promotes ECM stiffness [106]. *MCHR1*, a plasma membrane protein that inhibits cAMP accretion and induces intracellular calcium flux, was exclusively downregulated in AA-SScL fibroblasts, a finding that contrasts with the upregulation of *MCHR1* observed in IPF [107], which has pathologic features overlapping with SSc-PF.

*NEDD9* upregulation and *SOX5* downregulation contributed to the functional enrichment of the Biological Process “Regulation of cell development” by ToppFun in AA-SScL. SOX5 is a transcription factor that is essential for chondrogenesis [108] and involved in lung development. Specifically, *Sox5^−/−^* mice exhibit lung abnormalities and reduced fibronectin expression [109]. SOX5 has been studied in diseases other than SSc. For example, in human mesenchymal stem cells, SOX5 contributed to the pathology of postmenopausal osteoporosis (PMOP), and its silencing increased collagen I and decreased KLF4 expression [110]. In fibroblast-like synoviocytes from rheumatoid arthritis patients, silencing SOX5 decreased *IL-6* expression and reduced *MMP-1*,-2, and -9 [111]. *SOX5* is expressed in human lung fibroblasts where it can be upregulated by endothelin-1 (ET-1); however, its role in fibroblasts and fibrosis has not been characterized. Given that SOX5 regulates genes in other cell types that are associated with SSc, SOX5 merits further investigation in lung fibrosis. 

To our knowledge, this is the first characterization of the unique disease mechanisms at play in AA-SScL fibroblasts and its resulting functional enrichment and impacted pathway analysis. The transcriptomic signature captured between SScL and NL fibroblasts in AA-SScL fibroblasts is intriguing and may hold the key to understanding racial disparity in SSc-PF.

### 3.4. The Unique Transcriptome of AA-SScL Fibroblasts (AA-SScL vs. EA-SScL)

We identified 384 DEGs in AA-SScL vs. EA-SScL. Ninety-eight of them are uncategorized genes, which presents an interesting opportunity for future investigation given that these genes are unknown but potentially involved in SSc-PF pathogenesis. The remaining 286 DEGs represented enriched pathways and ontologies related to the biosynthesis of steroid hormones, alcohol metabolic process, genes encoding ECM, beta1 integrin cell surface interactions, and neutrophil activation. 

One of the upregulated hit genes driving the “Cholesterol” and “Steroid biosynthetic” pathways was *DHCR7* (encoding for the enzyme 7-dehydrocholesterol reductase). This enzyme acts as a “switch” between cholesterol and vitamin D3 synthesis: DHCR7 can convert 7-dehydrocholesterol (7DHC) into cholesterol, and UVB radiation from sunlight converts 7DHC into vitamin D3, an essential steroid hormone involved in calcium and mineral metabolism, skeletal health, immune and cardiovascular functions [112,113,114]. Patwardhan et al. described a model in which DHCR7 and UVB photons compete for 7DHC substrate in the skin, leading individuals with high serum levels of DHCR7 to have poor vitamin D synthesis and high skin cholesterol levels for a given exposure to sunlight [115]. Vitamin D deficiency is a common factor in many autoimmune diseases [116], and nearly all AAs have vitamin D deficiency [117,118]. Interestingly, vitamin D3 and analogs have shown efficacy in the treatment of fibrotic skin conditions, including SSc, by inhibiting Th2 cytokine- and TGFβ-induced periostin expression [119]. In chronic vitamin D deficient mice, triggering of the renin-angiotensin system (RAS) led to activation of TGFβ1 and pulmonary fibrosis [120]. Treatment with vitamin D prevented lung fibrosis by blocking the TGFβ/Smad3 axis, repressing epithelia-mesenchymal transition (EMT), and restoring mRNA levels of the vitamin D receptor (VDR) [121,122,123]. 

Based on the impact analysis method that iPathwayGuide developed, we also identified the perturbation of pathways related to “Gap junction” and “Calcium signaling,” “GABAergic synapse” and “Neuroactive ligand-receptor interaction,” “Regulation of actin cytoskeleton,” “Hematopoietic cell lineage” and “Intestinal immune network for IgA production,” and “mRNA surveillance.” 

The enrichment of the “Gap junction” pathway, driven by the robust downregulation of *PDGFB* and the predicted increase in connexin genes via dampening of the MAPK signaling pathway, reflects a significant alteration of the connexin-mediated communication in AA-SScL fibroblasts, a signature associated with the inflammatory response and fibrosis [124]. Downregulation of *PDGFB* also contributed to the enrichment of the “Calcium signaling” pathway and partly to the deregulation of the “Regulation of actin cytoskeleton” pathway.

The combined downregulation of *GNB3* and *GABBR1* in AA-SScL led to the enrichment of the “GABAergic synapse” pathway. Interestingly, fibroblasts can be converted into GABAergic interneurons with functional synapses [125]. The role of the GABAergic synapse pathway in pulmonary fibrosis is unclear as it has been most studied in the central nervous system (CNS). However, GABA, a major inhibitory neurotransmitter that regulates the balance between excitatory and inhibitory circuits in the human nervous system, has been linked to the modulation of autoimmune inflammation. Specifically, GABAergic agents have been found to inhibit inflammatory cytokine production by antigen-presenting cells (APCs), including IL6 [126]. Likewise, GABA stimulation reduced IL6 and IL12 production by activated macrophages [127]. Immune cell-fibroblast cross-talk has been implicated in SSc, and IL6 expression is increased in SSc fibroblasts and exerts profibrotic effects [128,129]. Thus, there may be an unstudied link between GABAergic synapse pathways and inflammatory signaling in SSc. In addition, GABAergic signaling has been at the forefront of research during the Covid-19 pandemic because stimulation of the GABA type A receptors has been shown to prevent disease progression in COVID-19 patients [130]. Similarly, PABPC1 and PABPC4 have been recently identified as downregulated COVID-19-altered proteins, both of which are known autoantigens [131], offering a possible explanation for the autoimmune complications observed in COVID-19 patients. The downregulation of both *PABPC1L* and *PABPC4L* enriched the pathway “RNA degradation,” specifically in the branch of the pathway pertaining to mRNA surveillance. This pertains to an elaborate set of mechanisms that ensure gene expression is adapted to cellular needs, mRNAs are suitable for translation, and mRNA-containing premature termination codons are destroyed via nonsense-mediated decay [132]. Considering that about 45% of COVID-19 survivors have developed post-COVID-19 pulmonary fibrosis (PCPF) [133], there may be a link between the GABAergic and RNA degradation signaling pathways and SSc pulmonary fibrosis, which deserves further study. 

Taken together, with the lack of overlap in disease mechanisms, our data in SScL fibroblasts highlight that the SSc phenotype is different in AA and EA patients, emphasizing the need to develop customized therapies. 

## 4. Materials and Methods

### 4.1. Patient Information and Cell Culture of the Primary Human Pulmonary Fibroblasts

SSc patients and healthy donors were all females, with ages ranging from 33−63 years old (yo) for healthy donors (NL) and from 37−62 yo for SSc-PF patients (SSc) (Table S3). All SSc patients selected had pulmonary fibrosis (FVC < 70%) [134]. None of the EA SSc patients had pulmonary hypertension. However, in the AA SSc patients, 4 had a combination of pulmonary fibrosis and pulmonary hypertension (PA mean > 25 mm Hg in SSc-59, SSc-85, SSc-46, and SSc-77) [134]. Primary NL and SScL fibroblasts were used in passage 3 for RNAseq and qPCR and in passages 3–7 for immunoblotting. Fibroblasts were cultured in 10-cm dishes in complete DMEM media (Mediatech Inc., Manassas, VA, USA) with 10% FBS (Sigma-Aldrich, Saint Louis, MO, USA) and 1X Antibiotic/Antimycotic (ThermoFisher Scientific, Waltham, MA, USA) to confluency. Cells were washed with PBS before being scraped in Trizol for RNA extraction.

### 4.2. RNA Extraction and Preparation

Total RNA was extracted from fibroblasts using miRNeasy Mini Kit (Qiagen, Germantown, MD, USA) per manufacturer’s instructions, and RNA quality and quantity were assessed using a NanoDrop Lite spectrophotometer (ThermoFisher Scientific, Waltham, MA, USA). 

### 4.3. RNA Sequencing & Differential Expression Analysis

Total RNA samples were sent to Novogene Corporation Inc. (Sacramento, CA, USA) for RNAseq analysis. A detailed description of their methods can be found in Nguyen et al. [135]. Paired-end clean reads were aligned to the Homo sapiens grch38 reference genome using the Spliced Transcripts Alignment to Reference (STAR) software. Several DE analyses were carried out using DESeq2 according to the experimental design shown in Figure S1 [136]. The log2 fold change (log2FC) was estimated, and the false discovery rate (FDR) adjusted *p*-value (q-value) was calculated based on the Benjamini-Hochberg multiple testing adjustment procedure using DESeq2 for each gene. The criteria of significance to identify differentially expressed genes (DEGs) were set at q-value < 0.1 and log2FC > 0.6 for upregulated DEGs, and log2FC < −0.6 for downregulated DEGs. Heatmap was generated from the normalized counts of all genes with a q-value below 0.1.

### 4.4. Functional Enrichment and Impact Analysis

The DEGs generated by DESeq2 were imported into iPathwayGuide (Advaita), which provides an impact analysis method [137,138,139]. A detailed description of their methods can be found in Nguyen et al. [135]. A *p*-value < 0.05 was set to indicate a significant difference. 

### 4.5. Immunoblotting 

Whole-cell lysates were harvested in RIPA buffer with EDTA (Cat# BP-115D, Boston BioProducts, Inc., Milford, MA, USA), 1X Halt protease inhibitor (Cat# 78430, ThermoFisher Scientific, Waltham, MA, USA), and 1X sodium orthovanadate phosphatase inhibitor (Cat# 13721-39-6, ThermoFisher Scientific, Waltham, MA, USA). The total protein concentrations of cell lysates were determined using Pierce BCA Protein Assay Kit (Cat# 23225, ThermoFisher Scientific, Waltham, MA, USA). Equal protein amounts (10 µg) were prepared in Sample Buffer (Cat# B0007, ThermoFisher Scientific, Waltham, MA, USA) and loaded onto SDS-PAGE gels using the Bolt system from ThermoFisher Scientific according to the manufacturer’s protocol with the following reagents: 1. Bolt 4-12% Bis-Tris Plus gels (Cat# NW04120) 2. Bolt MES SDS Running buffer (Cat# B0002) 3. Bolt Antioxidant (Cat# BT0005). SDS-PAGE was performed. Proteins were transferred onto nitrocellulose membranes (Cat# 10600015, Cytiva, Marlborough, MA, USA) at 300 mA for 2 h before blocking in 5% non-fat dry milk (Carnation) in TBS-Tween-20 for one hour at room temperature. Blots were placed in primary antibody dilutions at 4 °C on a rotator overnight. All antibodies utilized are listed in Table S4. Blots were washed in TBS-Tween-20 the next day, placed in secondary antibody dilutions for one hour at room temperature, washed again, and imaged using SignalFire Plus ECL reagent (Cat#12630S, Cell Signaling, Danvers, MA, USA) or SuperSignal West Pico Plus (Cat# 34578, ThermoFisher Scientific, Waltham, MA, USA) on the FluorChem R imaging system (ProteinSimple, San Jose, CA, USA). Densitometry was performed using ImageJ software [140] and analyzed on GraphPad Prism version 9.4.0 (GraphPad Software Inc., La Jolla, CA, USA). 

### 4.6. cDNA and qPCR

cDNA was synthesized from RNA extracted from human lung fibroblasts using the iScript cDNA Synthesis Kit (Cat# 1708891, Bio-Rad, Hercules, CA, USA) according to the manufacturer’s instructions. We used 1 μg of RNA per 20 μL cDNA reaction. cDNA was synthesized on C1000 Touch Thermal Cycler (Bio-Rad, Hercules, CA, USA) with the following protocol: A. Priming stage for 5 min at 25 °C. B. Reverse Transcription stage for 20 min at 46 °C. C. RT inactivation stage for 1 min at 95 °C. D. Hold at 4 °C.

The following reaction mixes were used for qPCR: A. 5 μL of TaqMan gene expression master mix (Cat# 4369016, ThermoFisher Scientific, Waltham, MA, USA) B. 3.5 μL of UltraPure DNase/RNase-Free Distilled Water (Cat# 10977015, ThermoFisher Scientific, Waltham, MA, USA) C. 0.5 μL of GAPDH and B2M for duplexed housekeeping gene controls or D. 0.5 μL target FAM primer. One μL of cDNA was added to 9 μL of the aforementioned primer reaction mix. The reaction was performed on the StepOne Plus Real-time PCR machine by Applied Biosystems (ThermoFisher Scientific, Waltham, MA, USA) using the following protocol: Holding stage (1) 15 min at 48 °C, (2) 10 min at 95 °C. Cycling Stage (1) 1 min at 95 °C, (2) 1 min at 60 °C for a total of 40 cycles. The target gene 2^−ΔCT^ values normalized to *GAPDH* or *B2M* were graphed and statistically analyzed in GraphPad Prism version 9 (GraphPad Software Inc., La Jolla, CA, USA). All qPCR primers utilized are listed in Table S5.

### 4.7. Statistical Analysis

All continuous variables were expressed as the mean ± standard deviation. All statistical analyses were done using GraphPad Prism version 9 for Windows (GraphPad Software Inc., La Jolla, CA, USA). An unpaired *t*-test was used for comparison. All *p*-values < 0.05 were considered statistically significant. Outlier points identified with the ROUT test (Q = 1%) were removed from the datasets. 

## 5. Conclusions

African ancestry is associated with a higher incidence of SSc than European ancestry, and AA patients experience more severe symptoms and higher mortality [9], yet few research efforts have focused on the cellular factors underlying severe disease in AA patients. To our knowledge, this is the first characterization of the transcriptome of lung fibroblasts of SSc patients and healthy donors of African ancestry. Our data highlight differences in disease mechanisms between AA and EA SScL fibroblasts and suggest that AA-NL fibroblasts are in a “pre-fibrosis” state, poised to respond to potential fibrotic triggers. The DEGs and pathways identified in our study provide a wealth of new information to better understand the racial disparity in SSc-PF and design novel targets for the development of more effective and customized therapies. 

The clinical implication of this study is the recognition that new therapies need to consider the ancestry and ethnicity of the patients to be personalized and more effective. Mechanisms mediating a multi-system disease such as SSc are complex. Our study does not address the potential role of biologically active protein cleavage products such as matrikines [141] or the role of extracellular vesicles [142,143] in promoting cell-to-cell communication. However, our findings provide valuable insights into molecular alterations that differentiate fibroblasts from lung tissues of AA in health and disease. 

## Figures and Tables

**Table 1:** Functional enrichment driven by down- and upregulated DEGs in AA-NL fibroblasts. Based on the results of the comparison AA-NL vs. EA-NL, all 33 downregulated DEGs (in blue) were entered in ToppFun to determine pathways (PW) and gene ontology terms, such as biological process (BP) and molecular function (MF). The same was performed for all 36 upregulated DEGs (in red). The *p*-values are shown in parentheses.

Enrichment	Hit in Query List
**Driven by downregulated DEGs**	**Downregulated DEGs entered**
PW- Beta1 integrin cell surface interactions (2.06 × 10^−8^) PW- Integrin signaling pathway (2.17 × 10^−6^) PW- Integrins in angiogenesis (2.53 × 10^−6^)	*COL3A1, COL5A1, COL5A2, LAMC1, COL1A1*
PW- Focal adhesion (5.13 × 10^−6^)	*COL3A1, COL5A1, COL5A2, LAMC1, COL1A1*
PW- Collagen biosynthesis and modifying enzymes (2.79 × 10^−8^) PW- Collagen formation (1.17 × 10^−7^) PW- ECM-receptor interaction (7.03 × 10^−8^) BP- ECM organization (2.01 × 10^−4^) PW- Genes encoding collagen proteins (3.06 × 10^−7^) PW- Collagen chain trimerization (4.01 × 10^−7^) PW- Assembly of collagen fibrils and other multimeric structures (1.09 × 10^−6^) BP- Collagen fibril organization (1.29 × 10^−6^) MF- ECM structural constituent conferring tensile strength (3.86 × 10^−7^) PW- Ensemble of genes encoding core ECM including ECM glycoproteins, collagens, and proteoglycans (1.08 × 10^−6^) PW- Extracellular matrix organization (1.72 × 10^−6^) BP- Extracellular structure organization (5.99 × 10^−5^) PW- Ensemble of genes encoding ECM and ECM-associated proteins (2.28 × 10^−4^)	*COL3A1, COL5A1, COL5A2, P4HA2, COL1A1, LAMC1, RSPO4, ABCG1*
BP- Negative regulation of endodermal cell differentiation (1.12 × 10^−5^) BP- Skin development (3.57 × 10^−4^)	*COL3A1, PTGS1, COL1A1, COL5A1, COL5A2*
PW- Syndecan-1-mediated signaling events (3.68 × 10^−7^)	*COL3A1, COL5A1, COL5A2, COL1A1*
MF- Platelet-derived growth factor binding (7.81 × 10^−7^) PW- Platelet activation (5.70 × 10^−4^)	*COL3A1, COL5A1, COL1A1, PTGS1*
PW- COX reactions (1.36 × 10^−3^)	*PTGS1*
**Driven by upregulated DEGs**	**Upregulated DEGs entered**
MF- Histone methyltransferase activity (H3-K9 specific) (4.52 × 10^−5^) MF- Histone-lysine N-methyltransferase activity (8.34 × 10^−4^)	*PRDM8, PRDM16*
MF- Extracellular matrix binding (1.56 × 10^−3^)	*TINAGL1, ADAMTS5*
MF- N-acetyllactosaminide beta-1,6-N-acetylglucosaminyltransferase activity (2.80 × 10^−3^)	*GCNT2*
MF- Activating transcription factor binding (3.22 × 10^−3^)	*PRDM16, PITX2*
MF- mRNA 3′-UTR binding (3.50 × 10^−3^)	*RBMS3, RBM38*
MF- L27 domain binding (3.74 × 10^−3^)	*LIN7A*
BP- Positive regulation of myoblast proliferation (4.13 × 10^−5^)	*MEIS2, PITX2*
BP- Sensory organ development (1.84 × 10^−4^)	*PRDM16, MEIS2, LIN7A, PITX2, EPHB1*
BP- Central nervous system projection neuron axonogenesis (2.81 × 10^−4^)	*PRDM8, EPHB1*

**Table 2:** Functional enrichment driven by DEGs exclusively deregulated in AA-SScL fibroblasts. The 275 DEGs exclusive to AA-SScL, shown in the blue section of the Venn diagram (Figure 3E), were entered in ToppFun for functional enrichment analysis. Below are pathways (PW) and molecular function (MF) ontologies with the lowest *p*-value and the most hit genes in the query list. Red: upregulated genes, blue: downregulated genes. Pathways identified by iPathwayGuide’s impact analysis are underlined. The *p*-values are shown in parentheses.

Enrichment	Hit in Query List
PW: ECM organization (6.66 × 10^−7^) PW: Ensemble of genes encoding ECM and ECM-associated proteins (1.00 × 10^−4^)	*ANXA5, ASPN, BMP5, CLEC14A, CLEC2B, COL1A2, COL6A6, EGFL7, FBLN1, FBLN2, ITIH5, KDR, LAMA2, MXRA5, NID1, OMD, P4HA2, S100A4, SEMA4D, SFRP2, SPARC, ADAMTS15, HPSE2, ITGA10, ITGB2, JAM2, LRP4, P4HA3, PARM1, SEMA3C, SEMA3D, TGFB3*
MF: Calcium ion binding (1.61 × 10^−6^)	*AIF1L, ANXA5, ASPH, ASPN, CD248, DLL4, EGFL7, FBLN1, FBLN2, FKBP9, FSTL5, NID1, PCDHGA4, PRRG3, RCN3, SPARC, SWAP70, S100A4, CAPS, CRACR2B, LRP4, PLCD4, PLN, PLS1, PRRG4*
BP: Calcium ion transport (3.35 × 10^−6^)	*ANXA5, ASPH, CACHD1, CLIC2, LHCGR, PANX1, TRPV4, CALCRL, CLDN16, CACNA1A, CRACR2B, CRH, DRD1, HES1, HOMER2, MCHR1, PLN, RAMP1, TRPC5*
BP: Cell adhesion (1.42 × 10^−9^)	*MYADM, ABAT, SWAP70, CYTH3, CD55, CD83, ACVRL1, NEDD9, BMP5, FBLN1, FBLN2, ACKR3, PKP2, NID1, KDR, GPNMB, SEMA4D, SFRP2, COL6A6, PCDHGA4, CARMIL1, LAMA2, XG, APOD, OMD, PDPN, TRPV4, DUSP10, EGFL7, PALLD, * *HLA-DPA1, MCAM, ITGB2, PGM5, JAM2, CADM1, NTM, HES1, SDK2, PODXL, ITGA10, ST6GAL1, NLGN1, CLDN16, EPHA4*
BP: Regulation of cell development (1.62 × 10^−8^)	*ATF5, BMP5, CARMIL1, DLL4, DUSP10, EPHB2, FBLN1, KDR, METRN, MME, MYADM, NEDD9, PDPN, SEMA4D, TRPV4, * *CACNA1A, EPHA4, EPOR, HES1, KALRN, LRP4, NGEF, NLGN1, NTM, OSTN, RAPGEF4, SEMA3C, SEMA3D, SOX11, SOX5, ST6GAL1, STX1B, TRPC5, XYLT1, ZNF536*
MF: Gated channel activity (1.90 × 10^−5^) MF: Passive transmembrane transporter activity (2.78 × 10^−5^)	*ASIC2, ASPH, CACHD1, CLCN4, CLIC2, EPHB2, GRIK2, HCN2, PANX1, PDPN, PKP2, SHROOM2, TRPV4, ANO5, AQP1, CACNA1A, CRH, KCNA4, KCNMB4, NLGN1, PLN, TRPC5*
PW: Insulin secretion (0.044) PW: Maturity onset diabetes of the young (0.007) PW: Type II diabetes mellitus (0.028)	*ADCY1, SLC2A1, CREB3L1, RAPGEF4, CAMK2B, KCNMB4* *HHEX, HES1* *MAPK10, IRS2, CACNA1A, SOCS2*
PW: TGF-beta signaling pathway (0.031)	*BMP5, GREM1, DCN, FBN1, PITX2, TGFB3, ACVR2B*
PW: Viral myocarditis (0.040)	*LAMA2, CD55,* *HLA-DPA1, ITGB2, CXADR*
PW: Proteoglycans in cancer (0.012)	*IGF2, KDR, TLR4, TWIST2, COL1A1, COL1A2, DCN, LUM, HPSE2, CAMK2B*

**Table 3:** Functional enrichment for the comparison “AA-SScL vs. EA-SScL.” Shown here are pathways (PW), biological process (BP), and molecular function (MF) ontologies with the lowest *p*-value and the most hit genes in the query list returned by ToppFun and iPathwayGuide (underlined). Red: upregulated genes, blue: downregulated genes. The *p*-values are shown in parentheses.

Enrichment	Hit in Query List
PW: Cholesterol biosynthetic (1.34 × 10^−6^) PW: Steroid biosynthetic (1.92 × 10^−6^) PW: Steroid biosynthesis (0.01558) PW: Activation of gene expression by SREBF (SREBP) (1.07 × 10^−4^)	*DHCR7, FASN, FDPS, LSS, MVD, NSDHL*
BP: Organic hydroxy compound metabolic process (1.66 × 10^−6^) BP: Alcohol metabolic process (7.24 × 10^−6^)	*DHCR7, FASN, FDPS, LSS, MVD, MYOF, NPC1, NPC2, NSDHL, SRD5A1, ADH6, ASAH2, CFTR, DRD4, GNB3, GRK3, MAOA, MC1R, P2RY1, SNCAIP, WNT4*
PW: Ensemble of genes encoding ECM and ECM-associated proteins (5.11 × 10^−4^)	*ANXA4, CSTB, CTSK, FGF5, OGN, PLXDC1, SPARCL1, TIMP2, ADAM32, ANGPTL1, CCL8, CHAD, COL5A3, COL7A1, COL11A2, CRISPLD1, CTSW, IL6, IL15, OGFOD2, PDGFB, SEMA3D, VWDE, WNT4*
PW: Neutrophil degranulation (9.41 × 10^−4^) BP: Neutrophil activation involved in immune response (2.68 × 10^−4^)	*ABCA13, CD59, CSTB, DNASE1L1, GM2A, NPC2, PTPRN2, TIMP2, VAT1*
PW: Beta1 integrin cell surface interactions (9.00 × 10^−4^)	*ITGA8, CD14, COL7A1, COL11A2, VCAM1*
PW: Gap junction (0.003218) PW: Calcium signaling (0.016369)	*PDGFB*
PW: GABAergic synapse (0.04595) PW: Neuroactive ligand-receptor interaction (0.029797)	*GNB3, GABBR1, DRD4, P2RY1, CALCRL, MC1R, RXFP1*
PW: Regulation of actin cytoskeleton (0.024435)	*PDGFB, MYH1, ITGA8, FGF5*
PW: Hematopoietic cell lineage (0.038564) PW: Intestinal immune network for IgA production (0.010566)	*HLA-DPA1, IL6, IL15, CD14,* *CD59*
PW: mRNA surveillance pathway (0.041045)	*PABPC4L, PABPC1L, DDX39B*

## Data Availability

The RNAseq data will be publicly available on NCBI GEO under access# GSE215841 after 31 December 2024.

## Supplementary Materials

The following supporting information can be downloaded at: https://www.mdpi.com/article/10.3390/ijms24043645/s1.
