# Supplementary material for: First Characterization of the Transcriptome of Lung Fibroblasts of SSc Patients and Healthy Donors of African Ancestry
Source: Int J Mol Sci. 2023 Feb 11;24(4):3645. doi: 10.3390/ijms24043645 (PMC9966000; doi:10.3390/ijms24043645)
Supplement: Supplementary file 1 [file ijms-24-03645-s001.zip › supp figures S1-S14.pptx]

## Slide 1
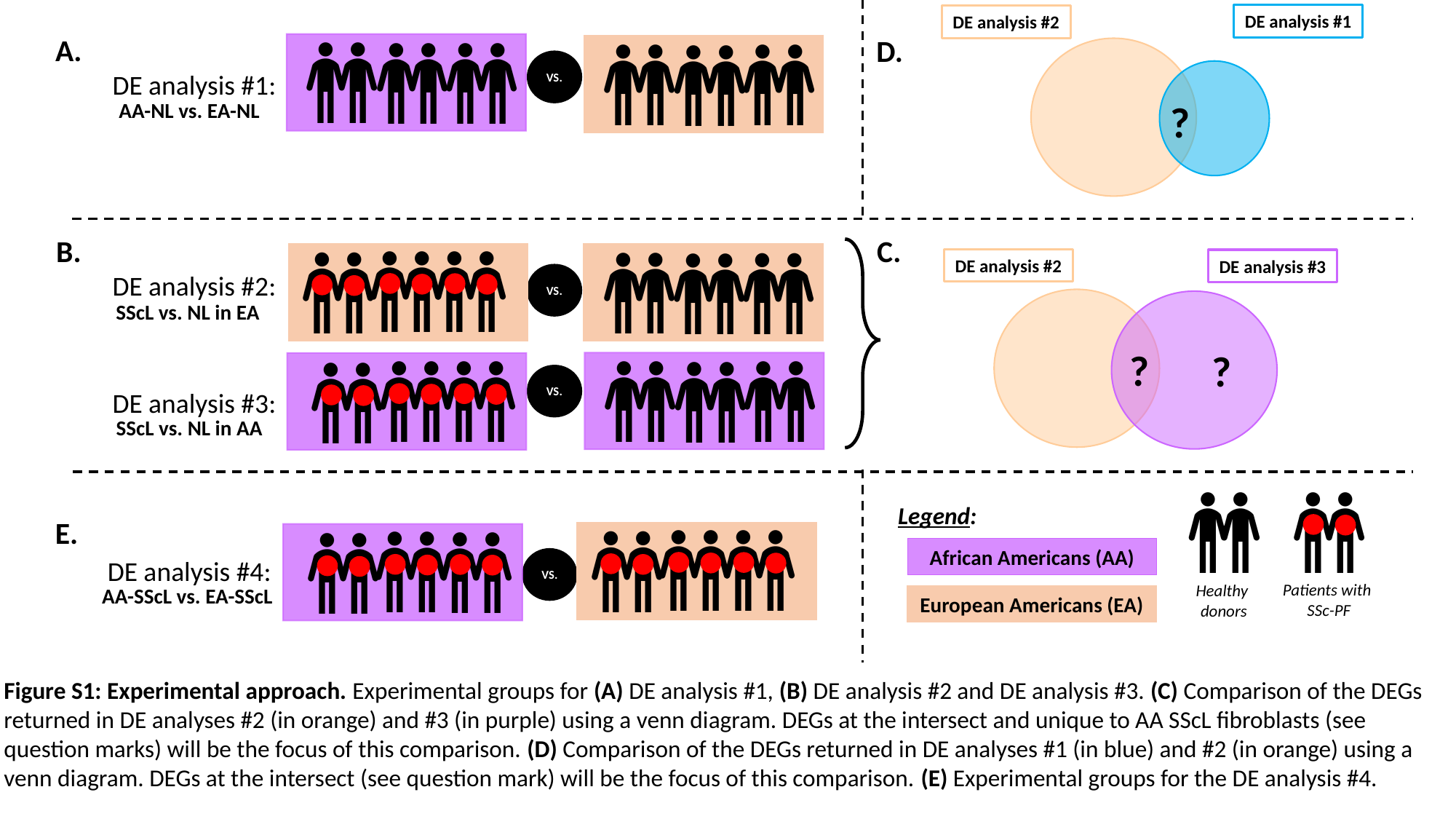

DE analysis #1
DE analysis #2
A.
D.
VS.
DE analysis #1:
?
AA-NL vs. EA-NL
B.
C.
DE analysis #2
DE analysis #3
DE analysis #2:
VS.
SScL vs. NL in EA
?
?
VS.
DE analysis #3:
SScL vs. NL in AA
Legend:
E.
African Americans (AA)
DE analysis #4:
VS.
Patients with
SSc-PF
Healthy
donors
AA-SScL vs. EA-SScL
European Americans (EA)
Figure S1: Experimental approach. Experimental groups for (A) DE analysis #1, (B) DE analysis #2 and DE analysis #3. (C) Comparison of the DEGs returned in DE analyses #2 (in orange) and #3 (in purple) using a venn diagram. DEGs at the intersect and unique to AA SScL fibroblasts (see question marks) will be the focus of this comparison. (D) Comparison of the DEGs returned in DE analyses #1 (in blue) and #2 (in orange) using a venn diagram. DEGs at the intersect (see question mark) will be the focus of this comparison. (E) Experimental groups for the DE analysis #4.

## Slide 2
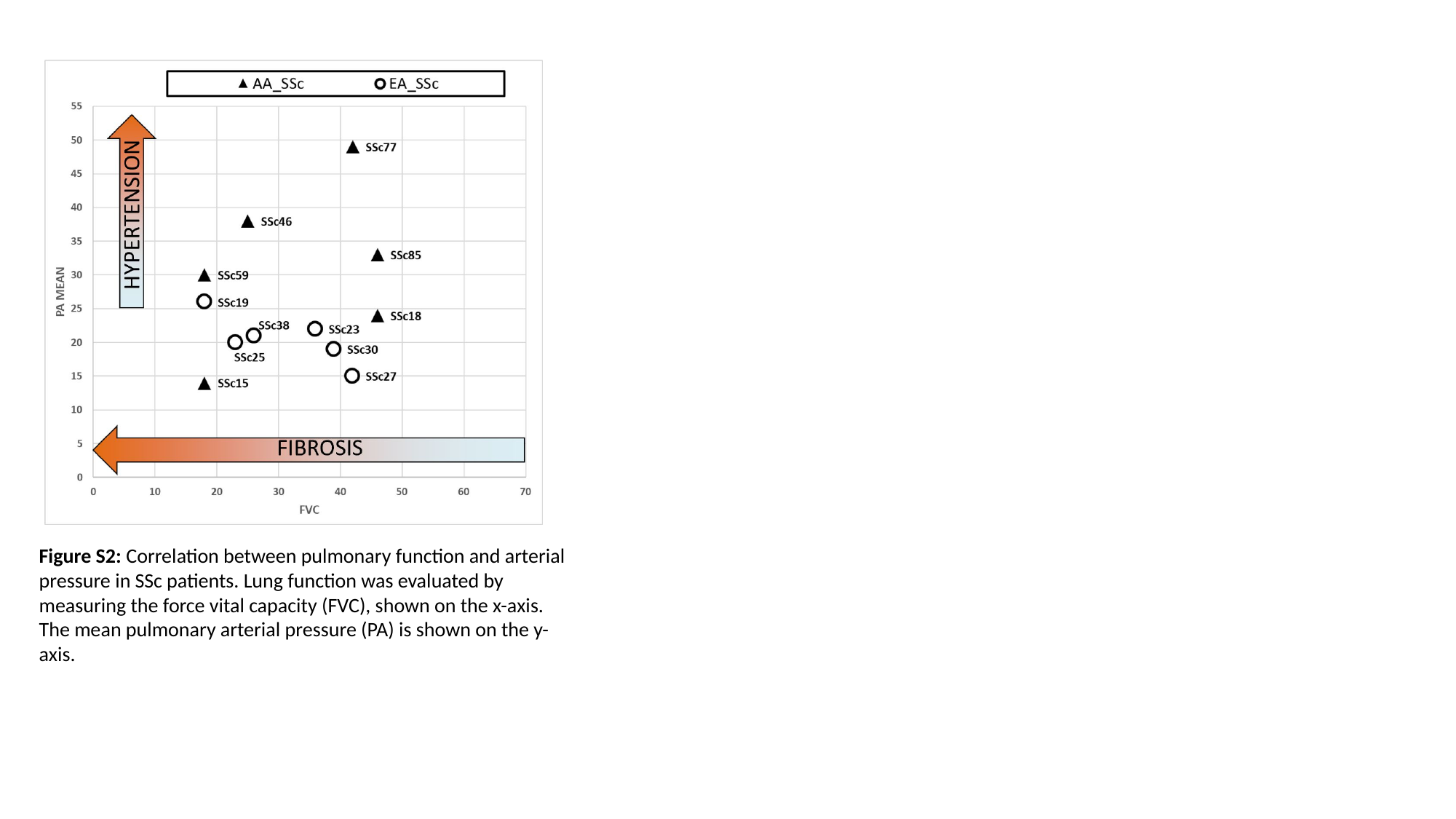

Figure S2: Correlation between pulmonary function and arterial pressure in SSc patients. Lung function was evaluated by measuring the force vital capacity (FVC), shown on the x-axis. The mean pulmonary arterial pressure (PA) is shown on the y-axis.

## Slide 3
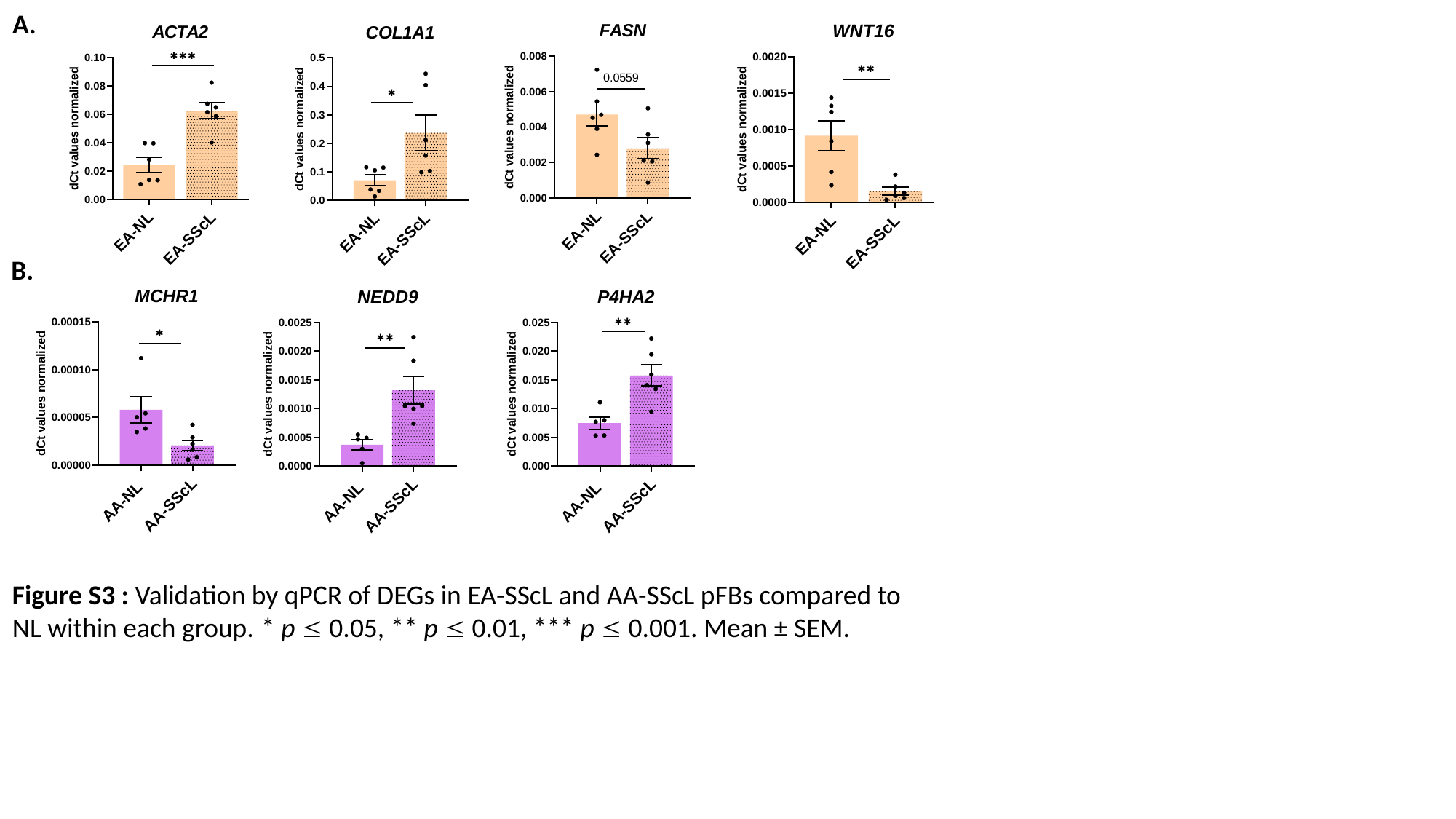

A.
B.
Figure S3 : Validation by qPCR of DEGs in EA-SScL and AA-SScL pFBs compared to NL within each group. * p  0.05, ** p  0.01, *** p ≤ 0.001. Mean ± SEM.

## Slide 4
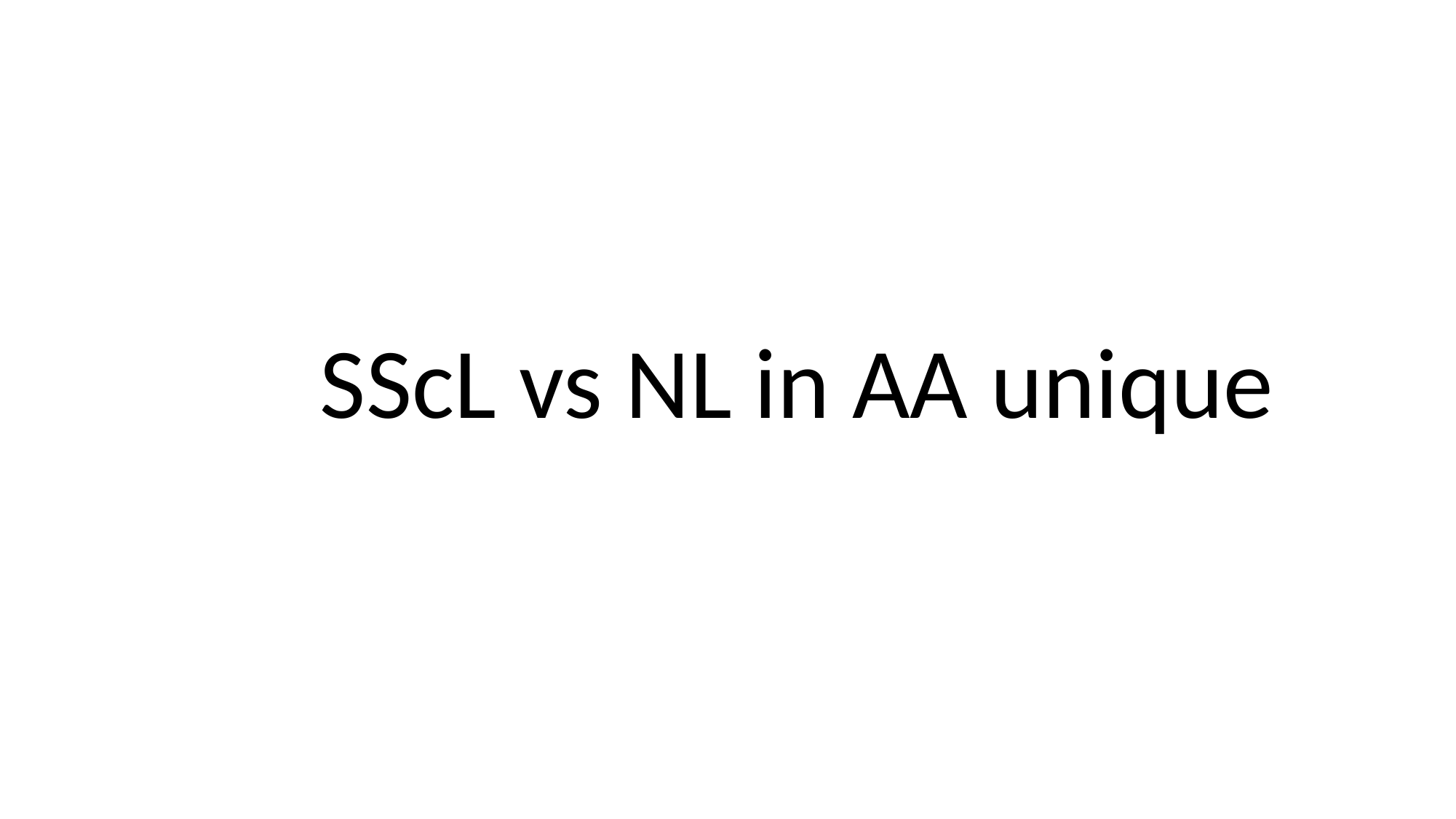

SScL vs NL in AA unique

## Slide 5
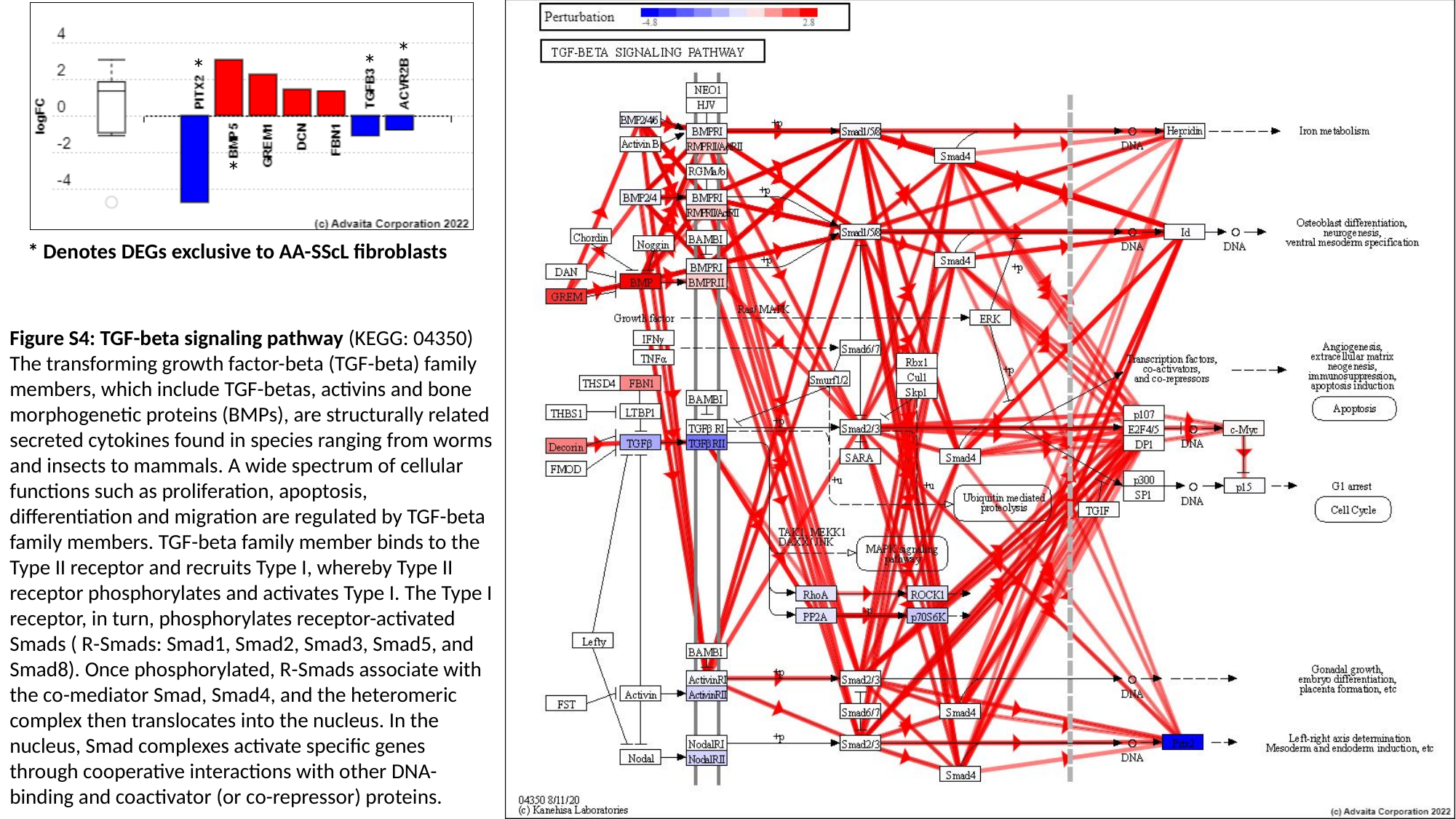

*
*
*
*
* Denotes DEGs exclusive to AA-SScL fibroblasts
Figure S4: TGF-beta signaling pathway (KEGG: 04350)
The transforming growth factor-beta (TGF-beta) family members, which include TGF-betas, activins and bone morphogenetic proteins (BMPs), are structurally related secreted cytokines found in species ranging from worms and insects to mammals. A wide spectrum of cellular functions such as proliferation, apoptosis, differentiation and migration are regulated by TGF-beta family members. TGF-beta family member binds to the Type II receptor and recruits Type I, whereby Type II receptor phosphorylates and activates Type I. The Type I receptor, in turn, phosphorylates receptor-activated Smads ( R-Smads: Smad1, Smad2, Smad3, Smad5, and Smad8). Once phosphorylated, R-Smads associate with the co-mediator Smad, Smad4, and the heteromeric complex then translocates into the nucleus. In the nucleus, Smad complexes activate specific genes through cooperative interactions with other DNA-binding and coactivator (or co-repressor) proteins.

## Slide 6
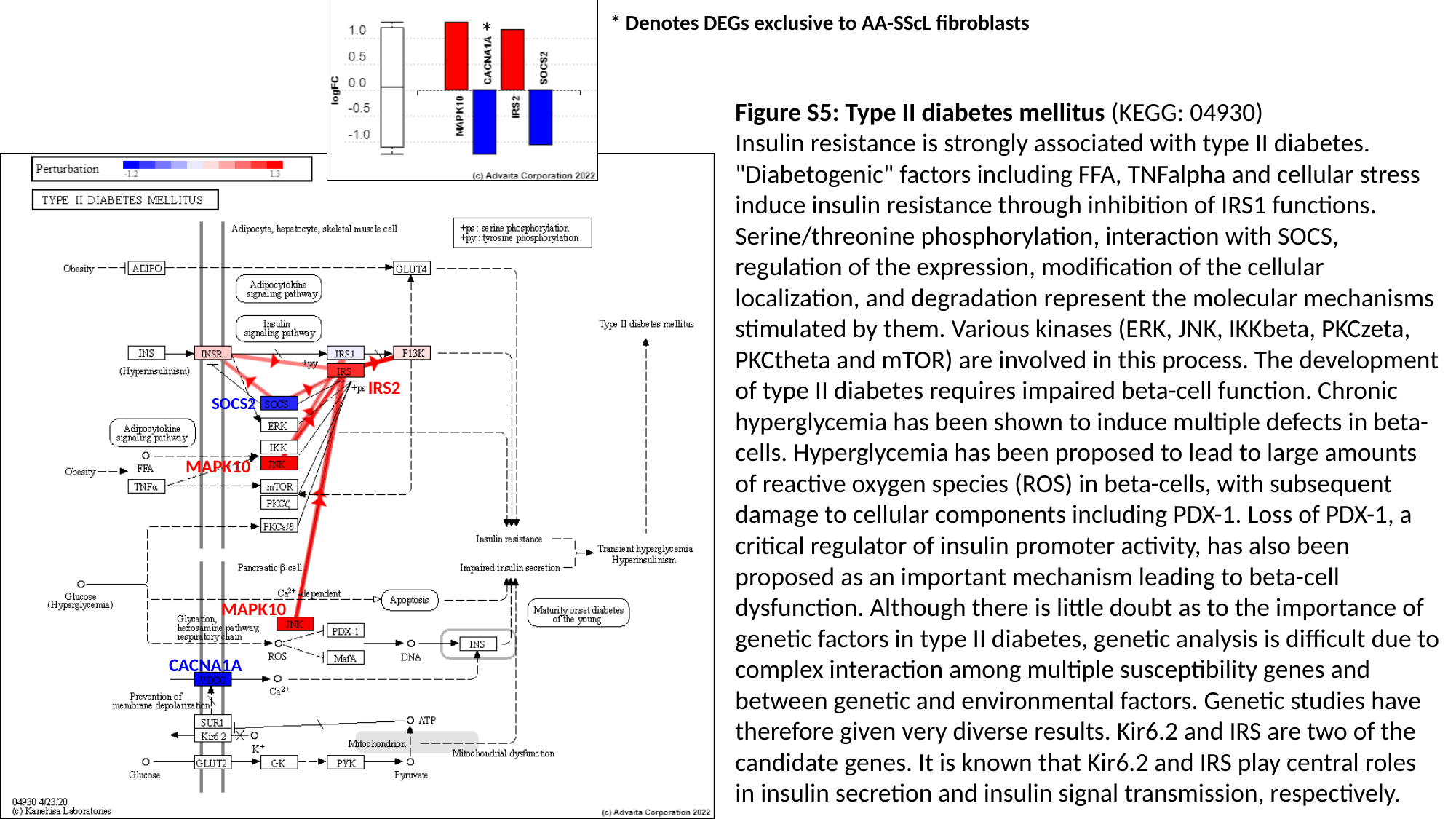

* Denotes DEGs exclusive to AA-SScL fibroblasts
*
Figure S5: Type II diabetes mellitus (KEGG: 04930)
Insulin resistance is strongly associated with type II diabetes. "Diabetogenic" factors including FFA, TNFalpha and cellular stress induce insulin resistance through inhibition of IRS1 functions. Serine/threonine phosphorylation, interaction with SOCS, regulation of the expression, modification of the cellular localization, and degradation represent the molecular mechanisms stimulated by them. Various kinases (ERK, JNK, IKKbeta, PKCzeta, PKCtheta and mTOR) are involved in this process. The development of type II diabetes requires impaired beta-cell function. Chronic hyperglycemia has been shown to induce multiple defects in beta-cells. Hyperglycemia has been proposed to lead to large amounts of reactive oxygen species (ROS) in beta-cells, with subsequent damage to cellular components including PDX-1. Loss of PDX-1, a critical regulator of insulin promoter activity, has also been proposed as an important mechanism leading to beta-cell dysfunction. Although there is little doubt as to the importance of genetic factors in type II diabetes, genetic analysis is difficult due to complex interaction among multiple susceptibility genes and between genetic and environmental factors. Genetic studies have therefore given very diverse results. Kir6.2 and IRS are two of the candidate genes. It is known that Kir6.2 and IRS play central roles in insulin secretion and insulin signal transmission, respectively.
IRS2
SOCS2
MAPK10
MAPK10
CACNA1A

## Slide 7
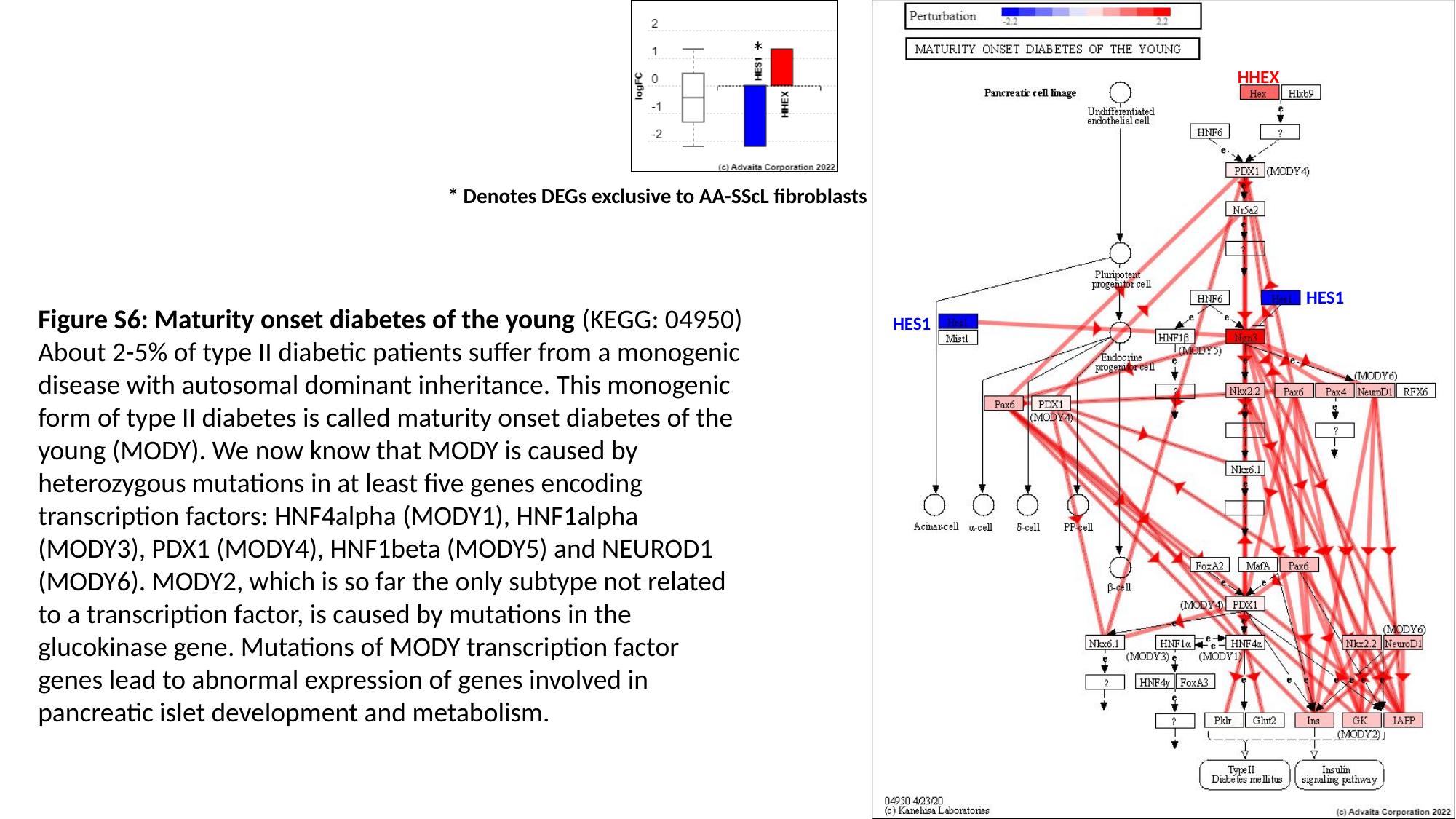

*
HHEX
* Denotes DEGs exclusive to AA-SScL fibroblasts
HES1
Figure S6: Maturity onset diabetes of the young (KEGG: 04950)
About 2-5% of type II diabetic patients suffer from a monogenic disease with autosomal dominant inheritance. This monogenic form of type II diabetes is called maturity onset diabetes of the young (MODY). We now know that MODY is caused by heterozygous mutations in at least five genes encoding transcription factors: HNF4alpha (MODY1), HNF1alpha (MODY3), PDX1 (MODY4), HNF1beta (MODY5) and NEUROD1 (MODY6). MODY2, which is so far the only subtype not related to a transcription factor, is caused by mutations in the glucokinase gene. Mutations of MODY transcription factor genes lead to abnormal expression of genes involved in pancreatic islet development and metabolism.
HES1

## Slide 8
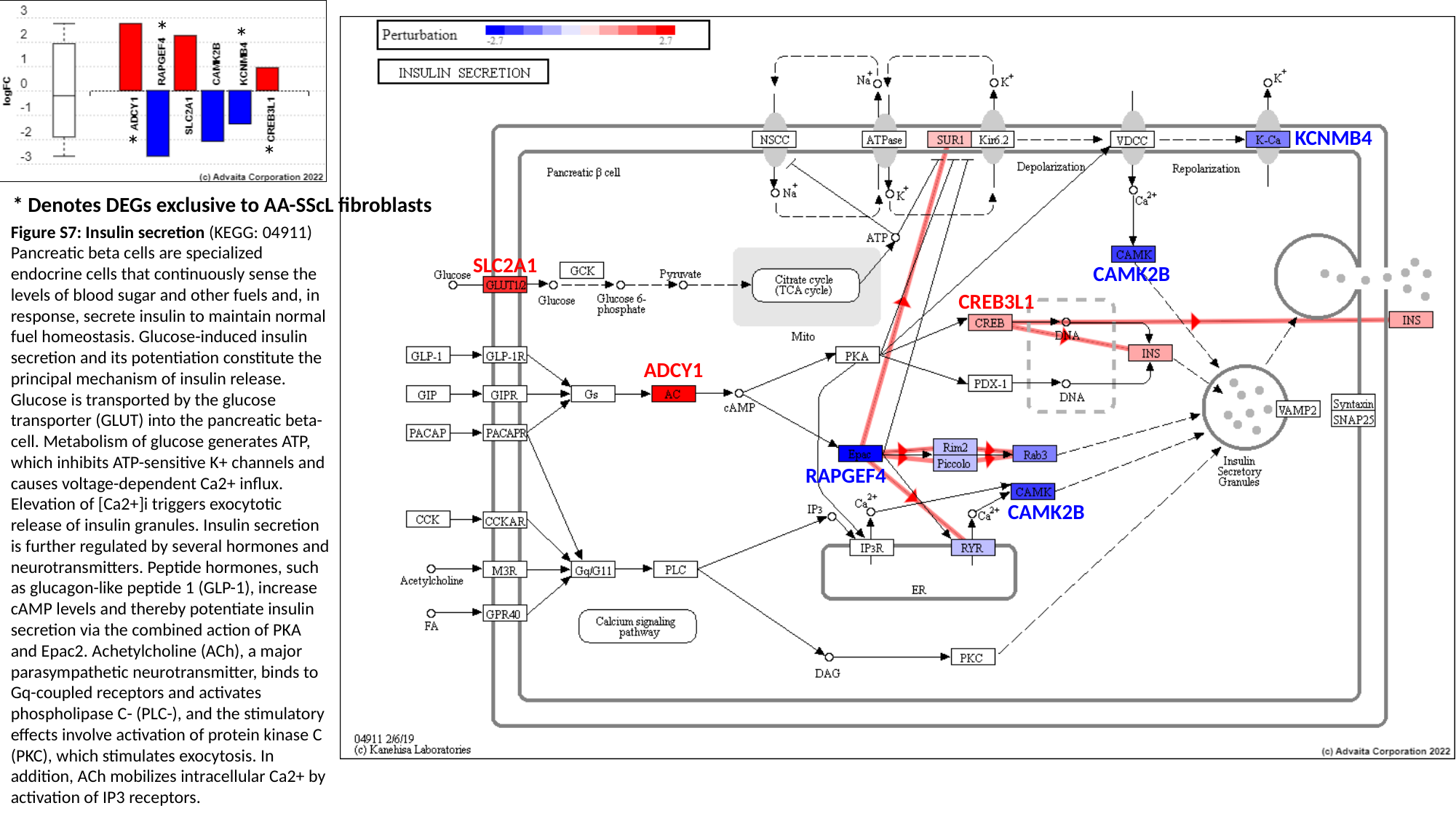

*
*
KCNMB4
*
*
* Denotes DEGs exclusive to AA-SScL fibroblasts
Figure S7: Insulin secretion (KEGG: 04911)
Pancreatic beta cells are specialized endocrine cells that continuously sense the levels of blood sugar and other fuels and, in response, secrete insulin to maintain normal fuel homeostasis. Glucose-induced insulin secretion and its potentiation constitute the principal mechanism of insulin release. Glucose is transported by the glucose transporter (GLUT) into the pancreatic beta-cell. Metabolism of glucose generates ATP, which inhibits ATP-sensitive K+ channels and causes voltage-dependent Ca2+ influx. Elevation of [Ca2+]i triggers exocytotic release of insulin granules. Insulin secretion is further regulated by several hormones and neurotransmitters. Peptide hormones, such as glucagon-like peptide 1 (GLP-1), increase cAMP levels and thereby potentiate insulin secretion via the combined action of PKA and Epac2. Achetylcholine (ACh), a major parasympathetic neurotransmitter, binds to Gq-coupled receptors and activates phospholipase C- (PLC-), and the stimulatory effects involve activation of protein kinase C (PKC), which stimulates exocytosis. In addition, ACh mobilizes intracellular Ca2+ by activation of IP3 receptors.
SLC2A1
CAMK2B
CREB3L1
ADCY1
RAPGEF4
CAMK2B

## Slide 9
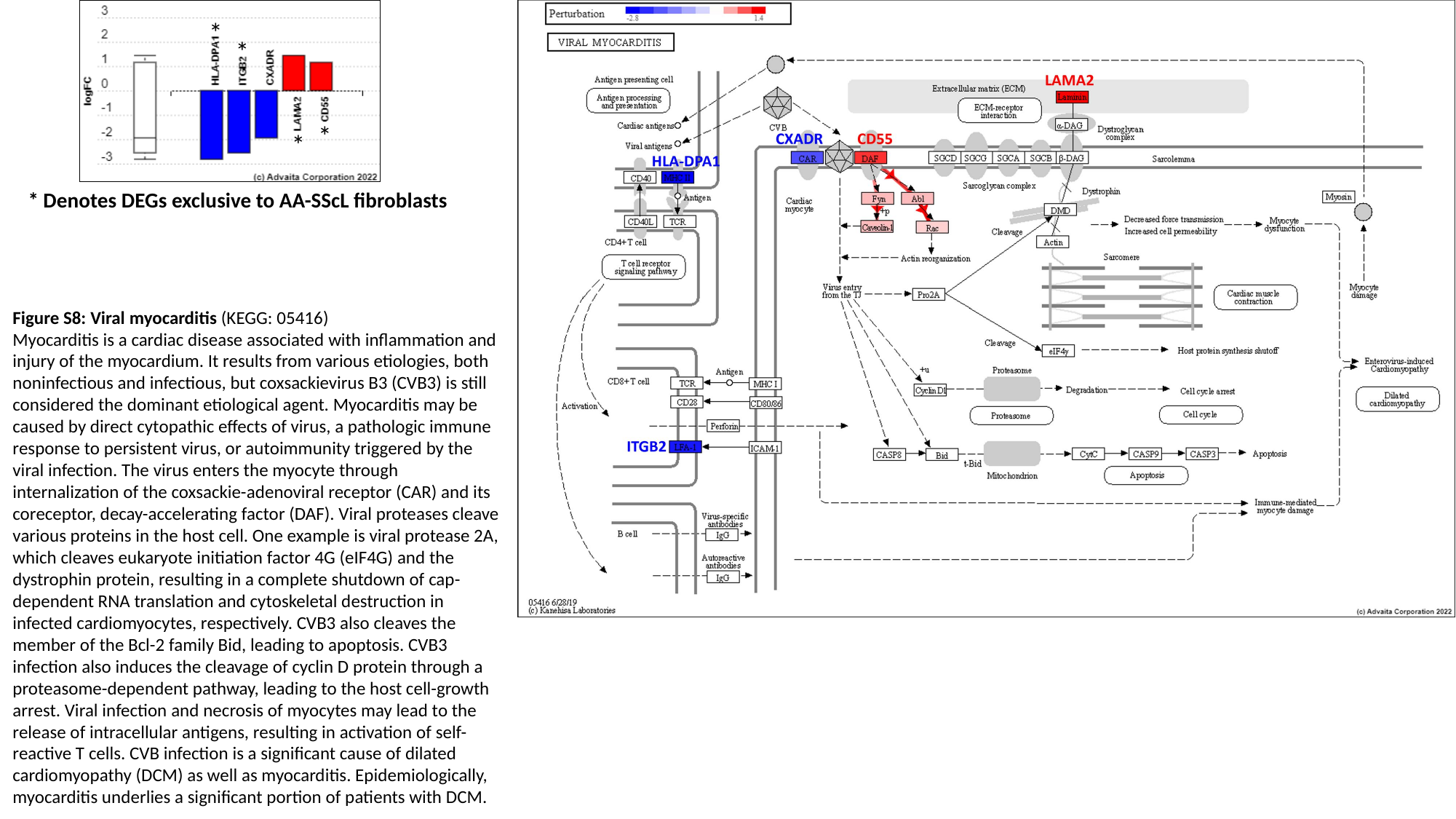

*
*
*
*
* Denotes DEGs exclusive to AA-SScL fibroblasts
Figure S8: Viral myocarditis (KEGG: 05416)
Myocarditis is a cardiac disease associated with inflammation and injury of the myocardium. It results from various etiologies, both noninfectious and infectious, but coxsackievirus B3 (CVB3) is still considered the dominant etiological agent. Myocarditis may be caused by direct cytopathic effects of virus, a pathologic immune response to persistent virus, or autoimmunity triggered by the viral infection. The virus enters the myocyte through internalization of the coxsackie-adenoviral receptor (CAR) and its coreceptor, decay-accelerating factor (DAF). Viral proteases cleave various proteins in the host cell. One example is viral protease 2A, which cleaves eukaryote initiation factor 4G (eIF4G) and the dystrophin protein, resulting in a complete shutdown of cap-dependent RNA translation and cytoskeletal destruction in infected cardiomyocytes, respectively. CVB3 also cleaves the member of the Bcl-2 family Bid, leading to apoptosis. CVB3 infection also induces the cleavage of cyclin D protein through a proteasome-dependent pathway, leading to the host cell-growth arrest. Viral infection and necrosis of myocytes may lead to the release of intracellular antigens, resulting in activation of self-reactive T cells. CVB infection is a significant cause of dilated cardiomyopathy (DCM) as well as myocarditis. Epidemiologically, myocarditis underlies a significant portion of patients with DCM.

## Slide 10
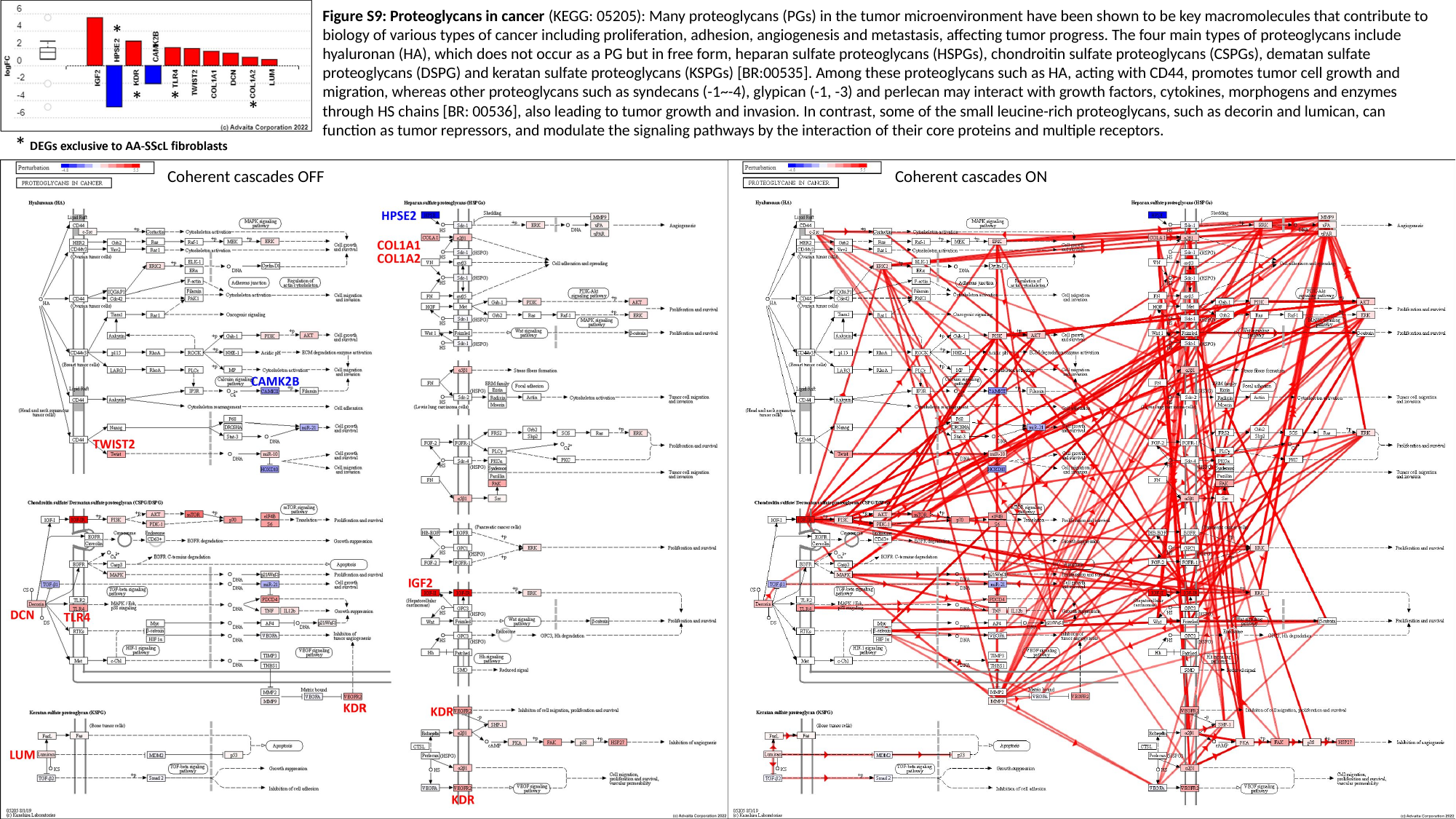

Figure S9: Proteoglycans in cancer (KEGG: 05205): Many proteoglycans (PGs) in the tumor microenvironment have been shown to be key macromolecules that contribute to biology of various types of cancer including proliferation, adhesion, angiogenesis and metastasis, affecting tumor progress. The four main types of proteoglycans include hyaluronan (HA), which does not occur as a PG but in free form, heparan sulfate proteoglycans (HSPGs), chondroitin sulfate proteoglycans (CSPGs), dematan sulfate proteoglycans (DSPG) and keratan sulfate proteoglycans (KSPGs) [BR:00535]. Among these proteoglycans such as HA, acting with CD44, promotes tumor cell growth and migration, whereas other proteoglycans such as syndecans (-1~-4), glypican (-1, -3) and perlecan may interact with growth factors, cytokines, morphogens and enzymes through HS chains [BR: 00536], also leading to tumor growth and invasion. In contrast, some of the small leucine-rich proteoglycans, such as decorin and lumican, can function as tumor repressors, and modulate the signaling pathways by the interaction of their core proteins and multiple receptors.
Coherent cascades OFF
Coherent cascades ON

## Slide 11
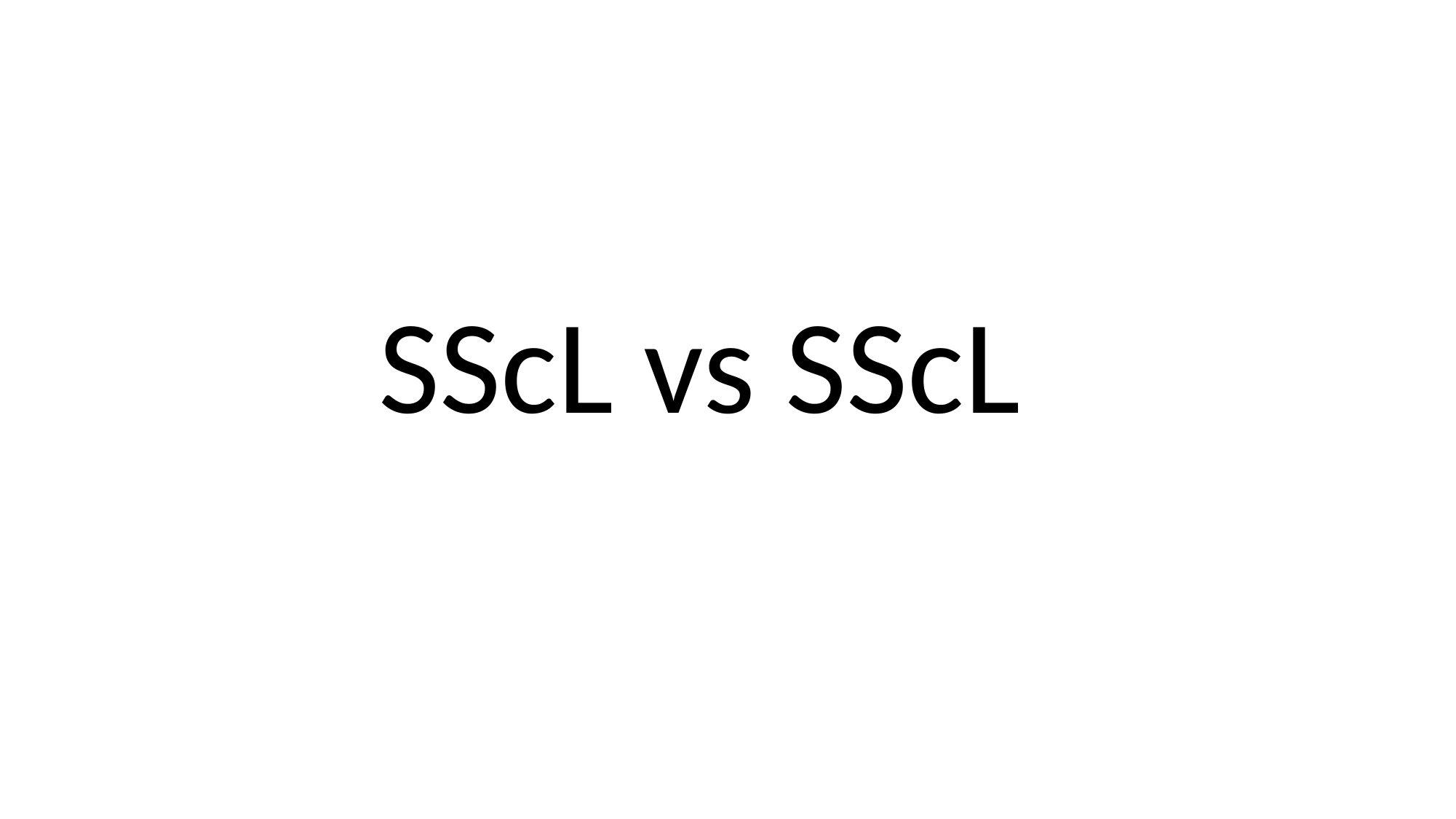

SScL vs SScL

## Slide 12
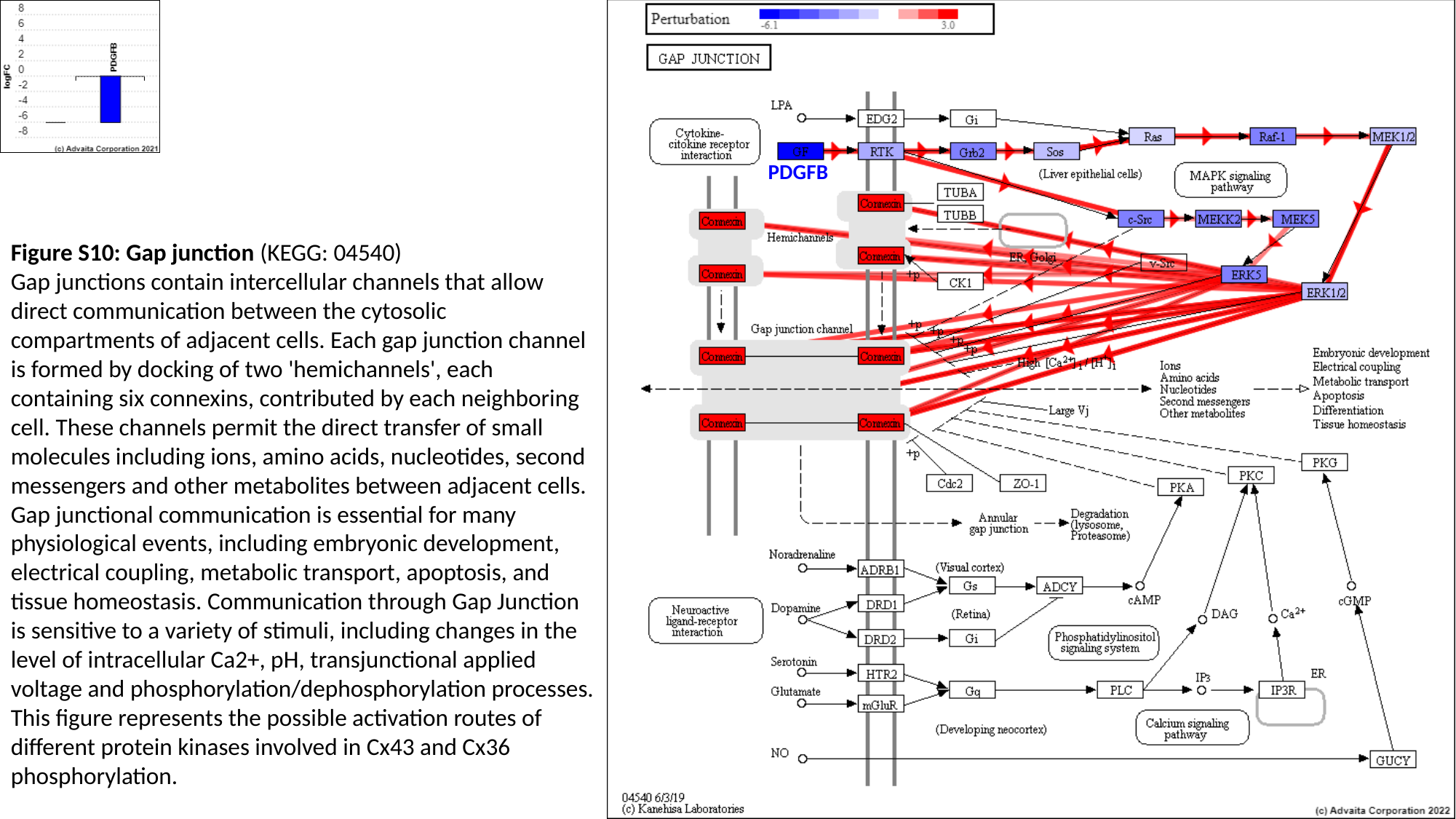

PDGFB
Figure S10: Gap junction (KEGG: 04540)
Gap junctions contain intercellular channels that allow direct communication between the cytosolic compartments of adjacent cells. Each gap junction channel is formed by docking of two 'hemichannels', each containing six connexins, contributed by each neighboring cell. These channels permit the direct transfer of small molecules including ions, amino acids, nucleotides, second messengers and other metabolites between adjacent cells. Gap junctional communication is essential for many physiological events, including embryonic development, electrical coupling, metabolic transport, apoptosis, and tissue homeostasis. Communication through Gap Junction is sensitive to a variety of stimuli, including changes in the level of intracellular Ca2+, pH, transjunctional applied voltage and phosphorylation/dephosphorylation processes. This figure represents the possible activation routes of different protein kinases involved in Cx43 and Cx36 phosphorylation.

## Slide 13
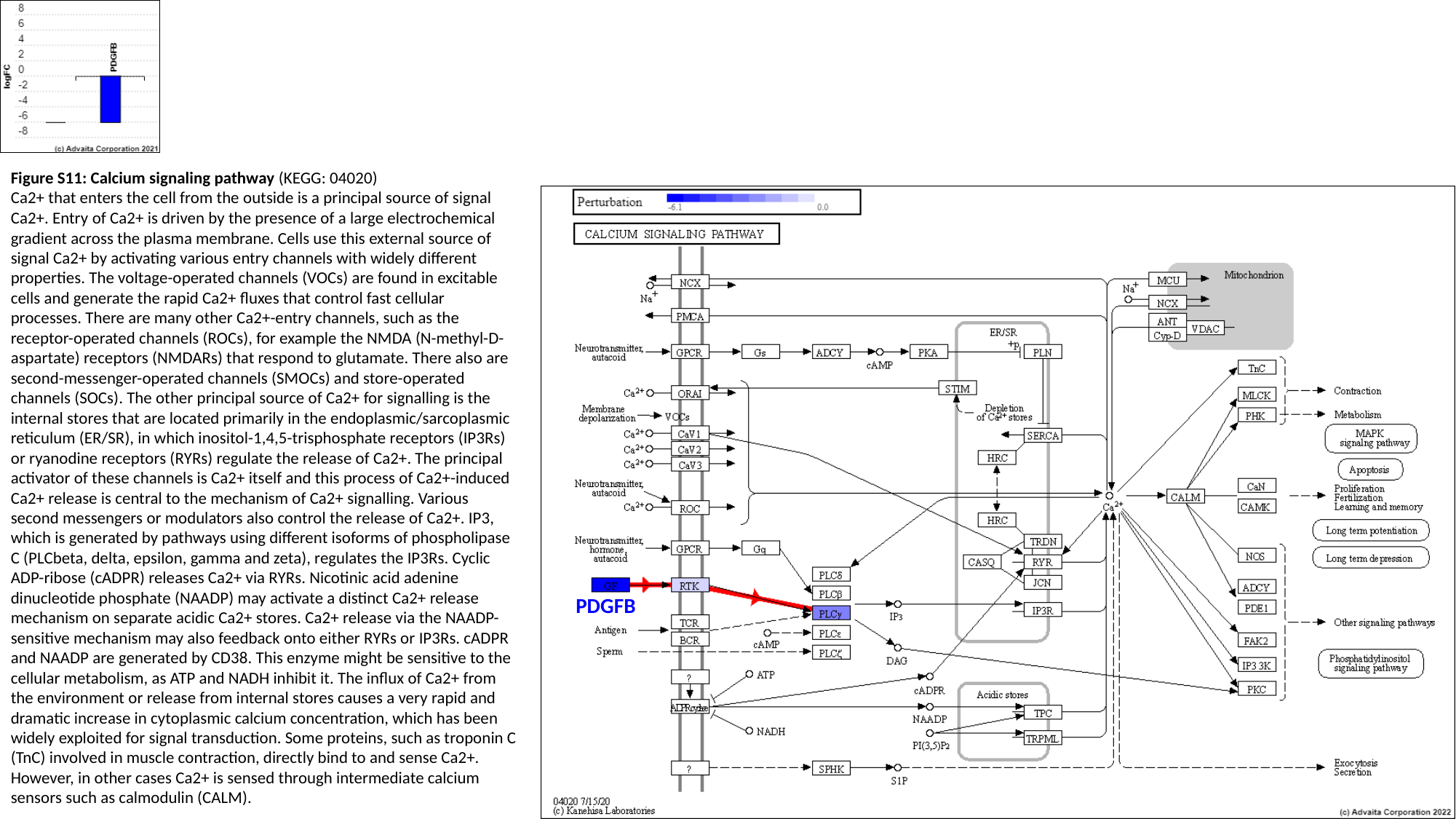

Figure S11: Calcium signaling pathway (KEGG: 04020)
Ca2+ that enters the cell from the outside is a principal source of signal Ca2+. Entry of Ca2+ is driven by the presence of a large electrochemical gradient across the plasma membrane. Cells use this external source of signal Ca2+ by activating various entry channels with widely different properties. The voltage-operated channels (VOCs) are found in excitable cells and generate the rapid Ca2+ fluxes that control fast cellular processes. There are many other Ca2+-entry channels, such as the receptor-operated channels (ROCs), for example the NMDA (N-methyl-D-aspartate) receptors (NMDARs) that respond to glutamate. There also are second-messenger-operated channels (SMOCs) and store-operated channels (SOCs). The other principal source of Ca2+ for signalling is the internal stores that are located primarily in the endoplasmic/sarcoplasmic reticulum (ER/SR), in which inositol-1,4,5-trisphosphate receptors (IP3Rs) or ryanodine receptors (RYRs) regulate the release of Ca2+. The principal activator of these channels is Ca2+ itself and this process of Ca2+-induced Ca2+ release is central to the mechanism of Ca2+ signalling. Various second messengers or modulators also control the release of Ca2+. IP3, which is generated by pathways using different isoforms of phospholipase C (PLCbeta, delta, epsilon, gamma and zeta), regulates the IP3Rs. Cyclic ADP-ribose (cADPR) releases Ca2+ via RYRs. Nicotinic acid adenine dinucleotide phosphate (NAADP) may activate a distinct Ca2+ release mechanism on separate acidic Ca2+ stores. Ca2+ release via the NAADP-sensitive mechanism may also feedback onto either RYRs or IP3Rs. cADPR and NAADP are generated by CD38. This enzyme might be sensitive to the cellular metabolism, as ATP and NADH inhibit it. The influx of Ca2+ from the environment or release from internal stores causes a very rapid and dramatic increase in cytoplasmic calcium concentration, which has been widely exploited for signal transduction. Some proteins, such as troponin C (TnC) involved in muscle contraction, directly bind to and sense Ca2+. However, in other cases Ca2+ is sensed through intermediate calcium sensors such as calmodulin (CALM).
PDGFB

## Slide 14
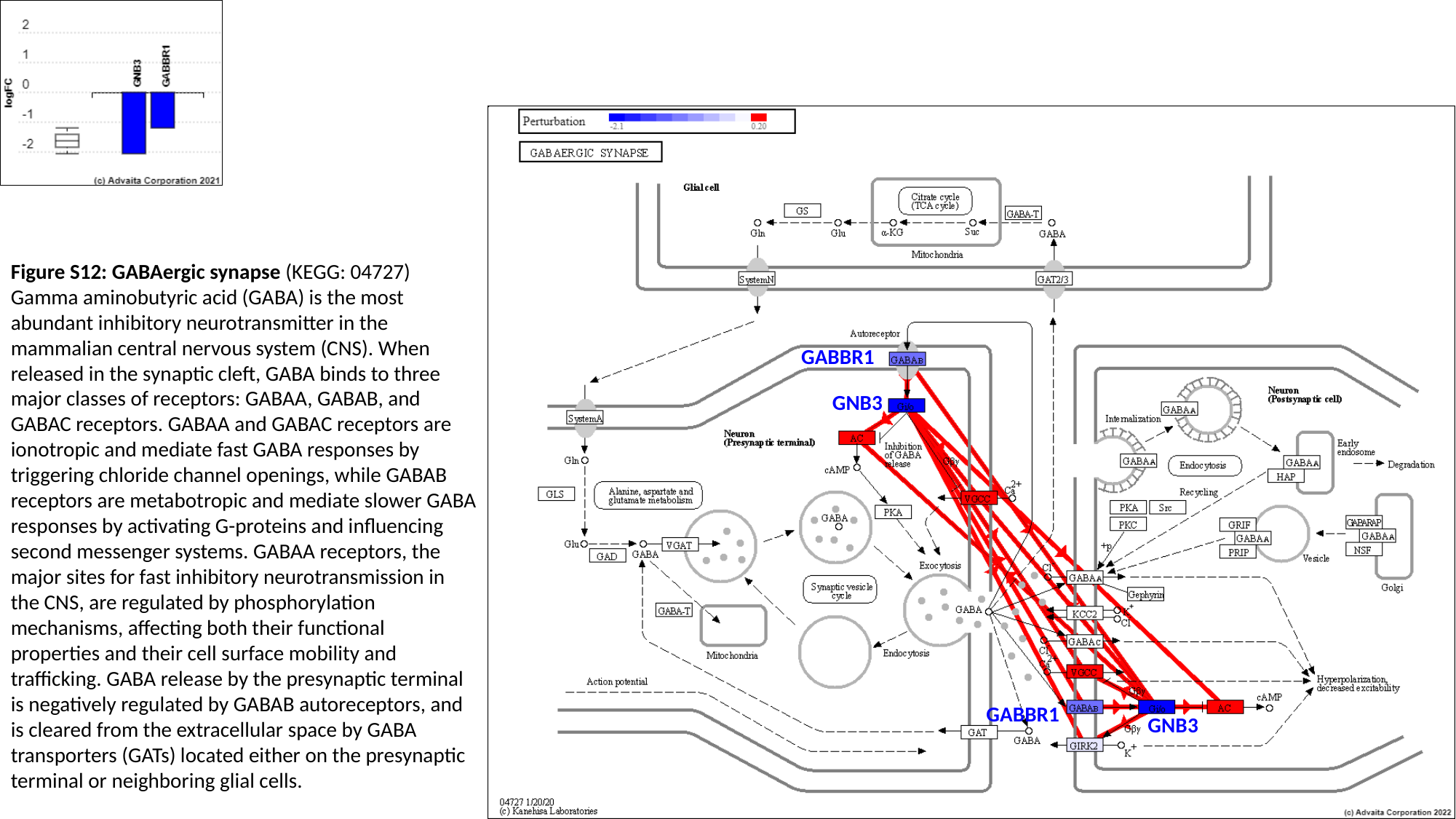

Figure S12: GABAergic synapse (KEGG: 04727)
Gamma aminobutyric acid (GABA) is the most abundant inhibitory neurotransmitter in the mammalian central nervous system (CNS). When released in the synaptic cleft, GABA binds to three major classes of receptors: GABAA, GABAB, and GABAC receptors. GABAA and GABAC receptors are ionotropic and mediate fast GABA responses by triggering chloride channel openings, while GABAB receptors are metabotropic and mediate slower GABA responses by activating G-proteins and influencing second messenger systems. GABAA receptors, the major sites for fast inhibitory neurotransmission in the CNS, are regulated by phosphorylation mechanisms, affecting both their functional properties and their cell surface mobility and trafficking. GABA release by the presynaptic terminal is negatively regulated by GABAB autoreceptors, and is cleared from the extracellular space by GABA transporters (GATs) located either on the presynaptic terminal or neighboring glial cells.
GABBR1
GNB3
GABBR1
GNB3

## Slide 15
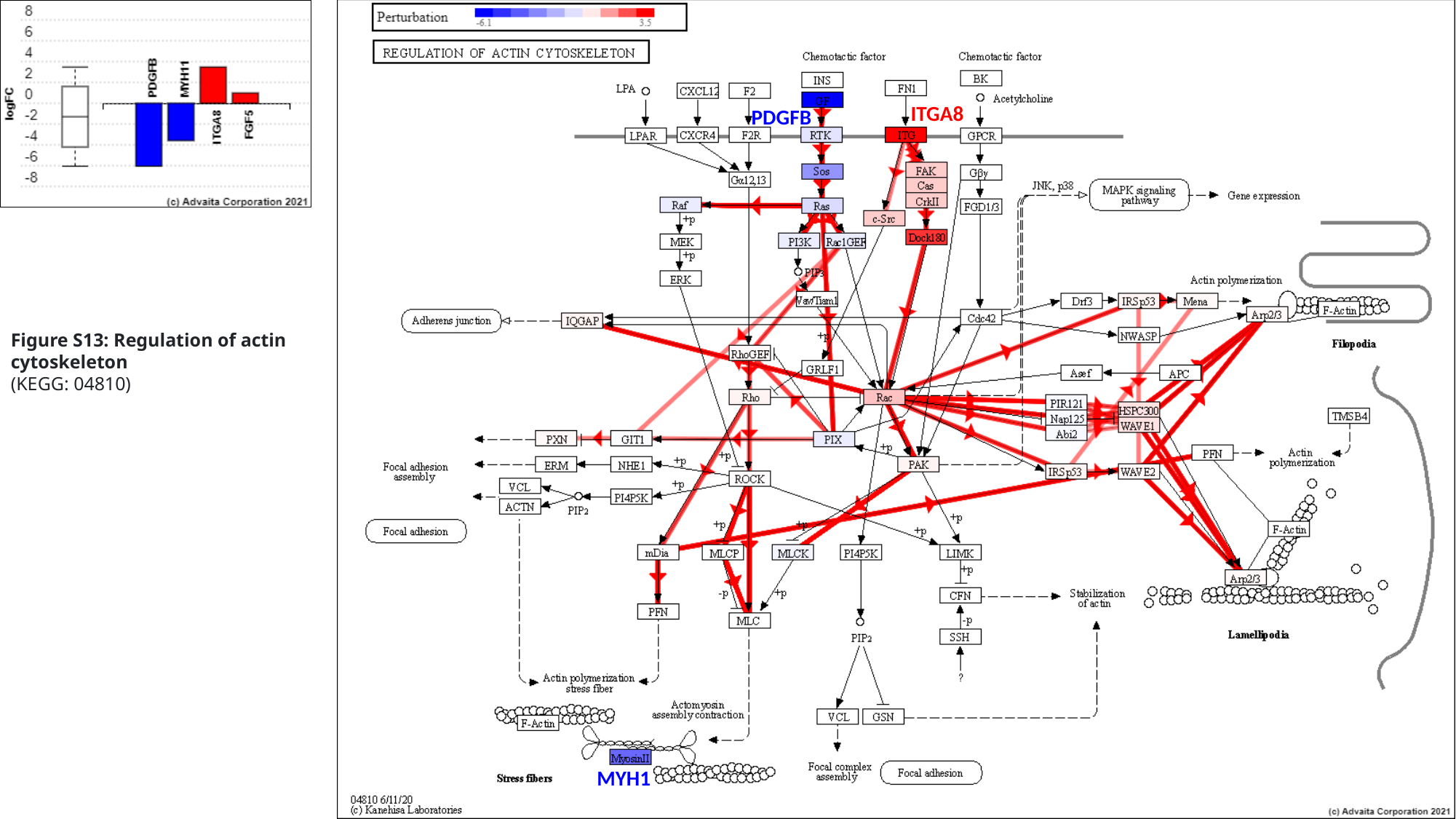

ITGA8
PDGFB
Figure S13: Regulation of actin cytoskeleton
(KEGG: 04810)
MYH1

## Slide 16
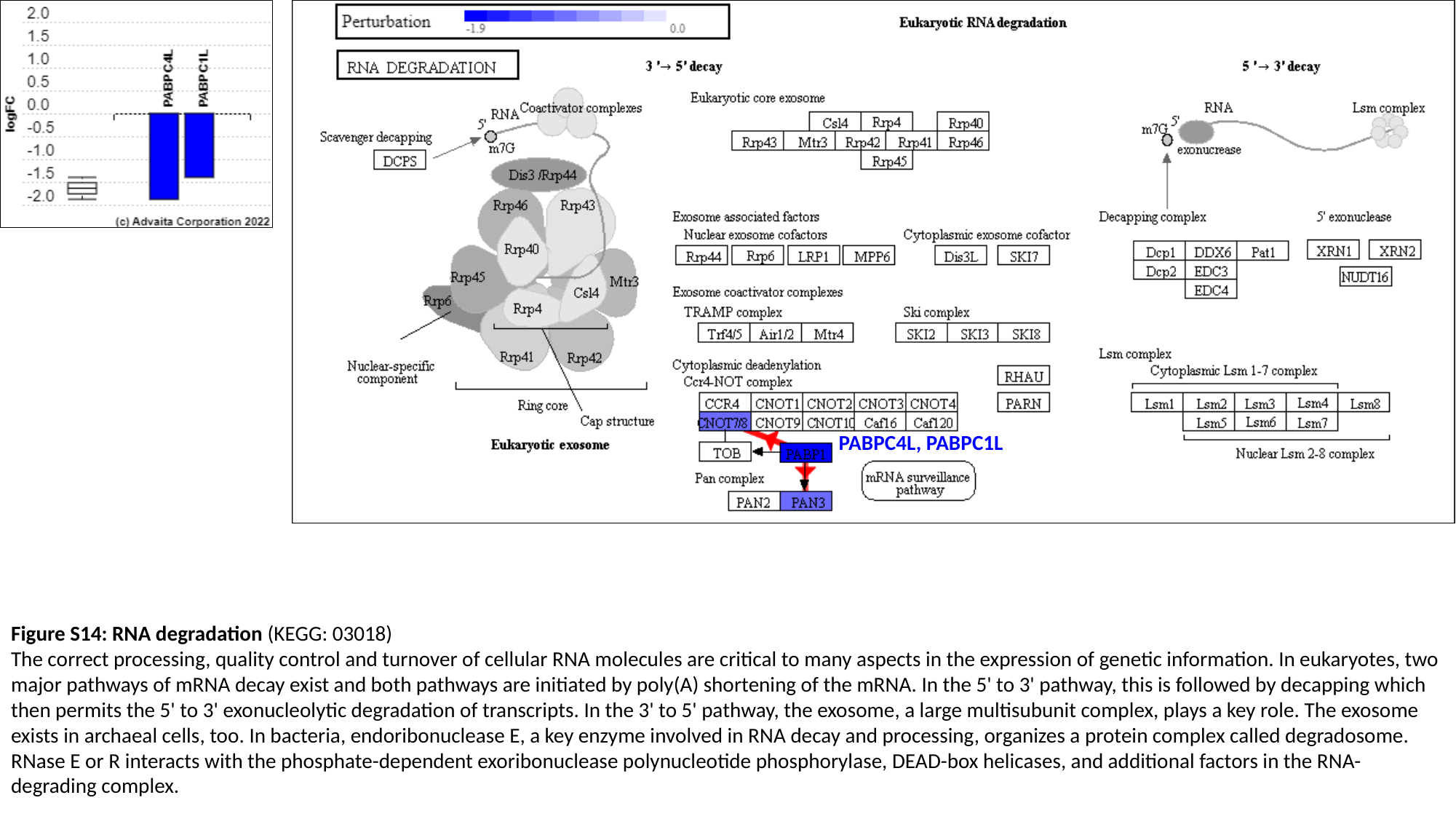

PABPC4L, PABPC1L
Figure S14: RNA degradation (KEGG: 03018)
The correct processing, quality control and turnover of cellular RNA molecules are critical to many aspects in the expression of genetic information. In eukaryotes, two major pathways of mRNA decay exist and both pathways are initiated by poly(A) shortening of the mRNA. In the 5' to 3' pathway, this is followed by decapping which then permits the 5' to 3' exonucleolytic degradation of transcripts. In the 3' to 5' pathway, the exosome, a large multisubunit complex, plays a key role. The exosome exists in archaeal cells, too. In bacteria, endoribonuclease E, a key enzyme involved in RNA decay and processing, organizes a protein complex called degradosome. RNase E or R interacts with the phosphate-dependent exoribonuclease polynucleotide phosphorylase, DEAD-box helicases, and additional factors in the RNA-degrading complex.
