# Supplementary material for: First Characterization of the Transcriptome of Lung Fibroblasts of SSc Patients and Healthy Donors of African Ancestry
Source: Int J Mol Sci. 2023 Feb 11;24(4):3645. doi: 10.3390/ijms24043645 (PMC9966000; doi:10.3390/ijms24043645)
Supplement: Supplementary file 1 [file ijms-24-03645-s001.zip › supp table S1_Results of the DE analysis AA-NL vs. EA-NL.docx]

**Table S1:** **Results of the DE analysis “AA-NL vs. EA-NL”.** Significantly upregulated genes are shown in red (q-value < 0.1, log2FC > 0.6) and significantly downregulated genes are shown in blue (q-value < 0.1, log2FC < -0.6). The table is sorted on “symbol” by alphabetical order.

| **symbol** | **log2FC** | **q-value** |
| --- | --- | --- |
| ABCG1 | -1.61681 | 0.0686 |
| AC004556.1 | -7.15972 | 0.062112 |
| AC006453.2 | 1.532451 | 0.003432 |
| AC008969.1 | 0.82277 | 0.080725 |
| AC026362.2 | 4.880805 | 0.075527 |
| AC138392.1 | 2.303908 | 0.001746 |
| AC237221.1 | 4.789963 | 0.003432 |
| ACTG2 | 1.337038 | 0.026904 |
| ADAMTS5 | 1.262017 | 0.08812 |
| AL117339.5 | 2.460763 | 0.005993 |
| AP003555.3 | 5.492201 | 0.080725 |
| BATF3 | -1.37332 | 0.000314 |
| CACHD1 | -0.82953 | 0.068515 |
| CAPS | 0.856863 | 0.084093 |
| COL1A1 | -0.9974 | 0.005594 |
| COL3A1 | -1.03874 | 0.021399 |
| COL5A1 | -0.90366 | 0.026904 |
| COL5A2 | -0.61658 | 0.097663 |
| CROCCP2 | 0.687661 | 0.025256 |
| EMP1 | -0.66122 | 0.0686 |
| EPHB1 | 2.396058 | 0.08812 |
| FAM198B-AS1 | -2.74862 | 0.00144 |
| FAM83H | -0.85304 | 0.047324 |
| FGD5 | -3.57737 | 0.0686 |
| FILIP1 | -2.03064 | 0.002623 |
| FLG-AS1 | 1.477031 | 0.007401 |
| FP671120.3 | 5.161184 | 0.006573 |
| GCNT2 | 1.146527 | 0.074931 |
| GFPT2 | -1.04234 | 0.098859 |
| H3F3AP4 | -1.4568 | 8.59E-07 |
| HAPLN1 | 5.090035 | 0.08565 |
| HLA-C | -1.14536 | 0.074931 |
| HMGB1P5 | 1.049328 | 0.068752 |
| HS3ST3A1 | -0.81877 | 0.018307 |
| HSPB7 | 2.097214 | 0.080725 |
| HTATIP2 | -0.74598 | 0.074931 |
| JPH2 | 1.949956 | 0.000173 |
| LAMC1 | -0.61765 | 0.074931 |
| LIN7A | 1.057477 | 0.007456 |
| LINC01013 | 4.382863 | 0.074931 |
| LINC01550 | 2.541408 | 0.097663 |
| MBNL1-AS1 | 0.722644 | 0.032269 |
| MEIS2 | 0.888747 | 0.0686 |
| MICA | -1.11435 | 0.000871 |
| MTND1P23 | 7.404502 | 8.67E-05 |
| NOTCH2NLA | 1.086819 | 0.068515 |
| P4HA2 | -0.72042 | 0.080725 |
| PCDHA10 | -1.62 | 0.084711 |
| PITX2 | 2.692833 | 0.003587 |
| PNMA2 | -2.15813 | 6.80E-07 |
| PNMA8A | 1.40957 | 0.08812 |
| PRDM16 | 1.784544 | 0.084456 |
| PRDM8 | 1.081328 | 0.08812 |
| PTGS1 | -1.08755 | 0.080725 |
| RASIP1 | -1.73418 | 0.005207 |
| RBM38 | 0.681776 | 0.080725 |
| RBMS3 | 0.624537 | 0.080921 |
| RPL10P9 | 4.644776 | 6.77E-07 |
| RPL9P9 | -0.77673 | 0.08985 |
| RSPO4 | -4.30375 | 0.010602 |
| SCN3A | 2.834411 | 0.08812 |
| SH3RF3-AS1 | -0.63112 | 0.074931 |
| SLC29A3 | -0.87082 | 0.094307 |
| SORD2P | 3.05546 | 0.000116 |
| STAC | -1.27823 | 0.003432 |
| TINAGL1 | 1.932274 | 0.0686 |
| TOX2 | -1.02131 | 0.001894 |
| TRABD2A | -0.90521 | 0.014042 |
| XCR1 | -4.05669 | 0.094307 |
