# Supplementary material for: First Characterization of the Transcriptome of Lung Fibroblasts of SSc Patients and Healthy Donors of African Ancestry
Source: Int J Mol Sci. 2023 Feb 11;24(4):3645. doi: 10.3390/ijms24043645 (PMC9966000; doi:10.3390/ijms24043645)
Supplement: Supplementary file 1 [file ijms-24-03645-s001.zip › supp table S2_Genes exclusively deregulated in AA-SScL pFBs.docx]

**Table S2: Genes exclusively deregulated in AA-SScL pFBs**. The genes listed here are from the venn diagram in Figure 3E in blue section, exclusively deregulated in AA-SScL fibroblasts. Red: upregulated in left column, and blue: downregulated in right column.

| **Upregulated** | **Downregulated** |
| --- | --- |
| ABAT | ABCB9 |
| ABCA9 | ABCD2 |
| AC004988.1 | AC010457.1 |
| AC005392.1 | AC018697.1 |
| AC011503.1 | AC026401.3 |
| AC098614.1 | AC083798.2 |
| AC245297.1 | AC116407.1 |
| ACAA2 | AC138866.2 |
| ACKR3 | AC156455.1 |
| ACVRL1 | ACP6 |
| ADCY1 | ACVR2B |
| AIF1L | ADAMTS15 |
| AK4P1 | ADHFE1 |
| AL450992.2 | ADORA2BP1 |
| ALDH1A3 | AFF2 |
| ANXA5 | AL157392.3 |
| APOD | AL354836.1 |
| ASIC2 | AL606760.1 |
| ASPH | AL683813.1 |
| ASPN | ALDH1A2 |
| ATF5 | ALPK3 |
| BAIAP2L2 | ANO5 |
| BASP1 | AQP1 |
| BMP5 | ASB5 |
| CABLES1 | BAALC |
| CACHD1 | C1orf74 |
| CARMIL1 | CA13 |
| CD163L1 | CACNA1A |
| CD248 | CADM1 |
| CD55 | CALCRL |
| CD83 | CAPS |
| CDC14B | CIITA |
| CES1 | CLDN16 |
| CHST7 | CRACR2B |
| CLCN4 | CRH |
| CLEC14A | CYP2S1 |
| CLEC2B | CYP3A5 |
| CLIC2 | DIRAS3 |
| COL1A2 | DOCK3 |
| COL6A6 | DOT1L |
| CREB3L1 | DPY19L2P2 |
| CYTH3 | DRD1 |
| DLL4 | EIF3FP3 |
| DUSP10 | EML6 |
| EGFL7 | ENAM |
| EMP1 | EPHA4 |
| EPHB2 | EPOR |
| EPS8L1 | FNBP1L |
| FAM174B | FP671120.4 |
| FAM198B-AS1 | GAP43 |
| FAM225A | GATD3B |
| FAM84A | GNAZ |
| FAM89A | HES1 |
| FBLL1 | HLA-DPA1 |
| FBLN1 | HOMER2 |
| FBLN2 | HPSE2 |
| FKBP9 | HSPB3 |
| FLT1 | IKZF2 |
| FSTL5 | IL31RA |
| GCSHP5 | INPP4B |
| GPNMB | ITGA10 |
| GPX7 | ITGB2 |
| GRIK2 | JAM2 |
| HCN2 | KALRN |
| HOXC9 | KCNA4 |
| HS3ST3B1 | KCNMB4 |
| IGDCC4 | KIAA0895L |
| INSYN1 | KRT32 |
| ITIH5 | LBX2 |
| JHY | LIN7A |
| KDR | LINC00327 |
| KIF17 | LINC00982 |
| LAMA2 | LINC01197 |
| LHCGR | LINC01569 |
| LINC00445 | LINC01679 |
| LINC00517 | LRCOL1 |
| LINC01094 | LRP4 |
| LINC01655 | MACROD2 |
| LINC01705 | MAST4 |
| LINC02257 | MCAM |
| LINC02421 | MCHR1 |
| LRRN4CL | MEDAG |
| METRN | MEF2C-AS1 |
| MME | MEIS2 |
| MSC | MEX3A |
| MSX2 | MIR137HG |
| MXRA5 | MIR31HG |
| MXRA5Y | MRM1 |
| MYADM | MYH11 |
| MYH13 | MYH15 |
| NDUFC2 | NFATC2 |
| NEDD9 | NGEF |
| NID1 | NLGN1 |
| NRIP1 | NTM |
| OMD | NUP210 |
| P4HA2 | OSTN |
| PALLD | P4HA3 |
| PANX1 | PARM1 |
| PAPSS2 | PGM5 |
| PCDHGA4 | PITX2 |
| PDE8A | PLCD4 |
| PDPN | PLIN4 |
| PFKFB3 | PLN |
| PGRMC1 | PLP1 |
| PIGZ | PLS1 |
| PKP2 | PODXL |
| PLEKHG1 | PRDM16 |
| PRRG3 | PRDM8 |
| PSG1 | PRRG4 |
| PTPRN2 | RAMP1 |
| RCN3 | RAPGEF4 |
| RGS7 | RTL4 |
| RPL3P4 | SCRG1 |
| S100A4 | SDK2 |
| SBF2-AS1 | SEMA3C |
| SEMA4D | SEMA3D |
| SEPTIN11 | SLC16A9 |
| SERINC2 | SOX11 |
| SFRP2 | SOX5 |
| SH3BP5 | SPAG17 |
| SHROOM2 | SPEG |
| SLC1A5 | ST6GAL1 |
| SLC36A1 | STEAP4 |
| SLC7A6 | STX1B |
| SNAI1 | TGFB3 |
| SOX13 | TMEFF2 |
| SPARC | TMSB15A |
| STK38 | TPTEP1 |
| SUDS3P1 | TRPC5 |
| SWAP70 | TSPAN2 |
| TBC1D2 | XYLT1 |
| TES | ZMAT1 |
| TLR4 | ZNF536 |
| TMEFF1 | ZNF620 |
| TMEM51 | ZNF883 |
| TRPV4 |  |
| ULBP2 |  |
| XG |  |
| XXYLT1-AS2 |  |
| ZSCAN31 |  |
